# A mammalian model reveals inorganic polyphosphate channeling into the nucleolus and induction of a hyper-condensate state

**DOI:** 10.1016/j.crmeth.2024.100814

**Published:** 2024-07-08

**Authors:** Filipy Borghi, Cristina Azevedo, Errin Johnson, Jemima J. Burden, Adolfo Saiardi

**Affiliations:** 1 Laboratory for Molecular Cell Biology, University College London, London WC1E 6BT, UK; 2 Sir William Dunn School of Pathology, University of Oxford, Oxford OX1 3RE, UK

**Keywords:** inorganic polyphosphates, liquid-liquid phase separation, polyphosphorilation, nucleus, nucleolus, toluidine blue, PPK1

## Abstract

Inorganic polyphosphate (polyP) is a ubiquitous polymer that controls fundamental processes. To overcome the absence of a genetically tractable mammalian model, we developed an inducible mammalian cell line expressing *Escherichia coli* polyphosphate kinase 1 (*Ec*PPK1). Inducing *Ec*PPK1 expression prompted polyP synthesis, enabling validation of polyP analytical methods. Virtually all newly synthesized polyP accumulates within the nucleus, mainly in the nucleolus. The channeled polyP within the nucleolus results in the redistribution of its markers, leading to altered rRNA processing. Ultrastructural analysis reveals electron-dense polyP structures associated with a hyper-condensed nucleolus resulting from an exacerbation of the liquid-liquid phase separation (LLPS) phenomena controlling this membraneless organelle. The selective accumulation of polyP in the nucleoli could be interpreted as an amplification of polyP channeling to where its physiological function takes place. Indeed, quantitative analysis of several mammalian cell lines confirms that endogenous polyP accumulates within the nucleolus.

## Highlights

• Development of the TREx-*Ec*PPK1 cell line to validate mammalian polyP research
• *Ec*PPK1-synthesized polyP exclusively localizes within the nucleus/nucleolus
• Endogenous polyP primarily localizes within the nucleolus
• Altering the LLPS properties of nucleolar polyP induces a hyper-condensed state

## Motivation

The processes regulated by polyP are numerous and varied, and some are directly linked to the pathophysiology of human diseases. However, a reliable model for manipulating polyP in mammalian cells is currently lacking. To provide researchers with such a model, we have developed an inducible cell line expressing *Escherichia coli* polyphosphate kinase 1 (*Ec*PPK1). These cells allowed the validation of polyP detection methods, the development of new assays, and the dissection of polyP-regulated physiology.

## Graphical abstract

**Figure:**
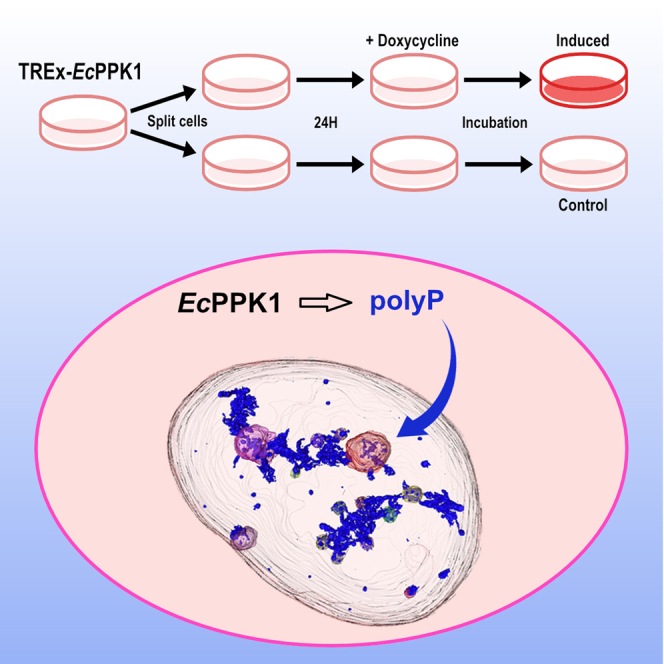

## Teaser

Inorganic polyphosphate (polyP), the simplest of biological polymers, is widely present in living organisms. Borghi et al. develop a cell line to provide researchers with a reliable model to dissect mammalian polyP physiology and unravel its potential role in human diseases.

## Introduction

The linear chain of phosphate (Pi) groups, referred to as inorganic polyphosphate (polyP), represents the simplest biological polymer. Due to its simplicity and ability to be synthesized under primordial Earth, polyP has been considered a “molecular fossil,” with very few roles in modern organisms. This perception is far from the truth. Over the past 20 years, research has demonstrated that polyP is widespread across biological systems from bacteria to humans.^1^^,^^2^^,^^3^^,^^4^ Organisms across various life kingdoms invest considerable genetic resources and biochemical energy in maintaining and regulating polyP metabolism, suggesting its essential biological utility. Indeed, polyP regulates fundamental physiological functions, from infectivity to chaperoning activity and the modulation of phosphate and cation homeostasis.^5^^,^^6^^,^^7^

PolyP metabolism is well understood in bacteria and microorganisms, with identified enzymes for its synthesis and degradation. Bacteria synthesize polyP using polyphosphate kinases (PPK1 and PPK2),^8^^,^^9^ while social amoebas utilize PPK1, acquired from bacteria,^10^ and yeast, *Trypanosomatida*, *Plasmodium*, and certain algae such as *Chlamydomonas* employ the vacuolar transporter chaperone complex to couple the synthesis and membrane translocation of the polymer.^11^^,^^12^^,^^13^ Knockouts of polyP synthesizing enzymes in these organisms have allowed the genetic validation of functions attributed to polyP. In higher eukaryotes, polyP synthesizing enzymes have not been discovered yet.^14^ Nevertheless, mammals seem to exhibit the most spectacular functions of this unique polymer; polyP controls blood coagulation cascade,^15^ Alzheimer’s neurotoxicity,^16^ and mitochondrial bioenergetics^17^ and contributes to motoneuron dysfunction.^18^

Despite prolific mammalian literature, genetic evidence is lacking, and some polyP detection assays have been revealed to be untrustworthy. One example is the widely used polyP-4′,6-diamidino-2-phenylindole (DAPI) red fluorescence shift. This shift is a feature shared by polyP-DAPI but also by the DAPI complex with RNA, amorphous calcium phosphate, and inositol phosphates.^19^^,^^20^ Another contentious issue is the extraction and biochemical analysis of polyP using polyacrylamide gel electrophoresis (PAGE). The use of non-standardized extraction and PAGE procedures has resulted in some laboratories being unable to purify and visualize mammalian polyP through PAGE. Our own laboratory, employing an extraction protocol validated for yeast and amoeba, failed to detect polyP in mammalian HTC116 cells using PAGE, even after [^32^P]orthophosphate radiolabeling.^21^

We developed a stable, inducible cell line expressing *E. coli*’s PPK1 gene (*Ec*PPK1) to validate mammalian polyP visualization methods, extraction procedures, and analysis protocols while also developing new analytical tools. Surprisingly, >95% of newly synthesized polyP selectively localizes in the nucleus, mainly in the nucleolus. Although the presence of polyP in the nucleolus, especially post-apoptosis, has been previously noted,^22^^,^^23^ its exclusive accumulation within this organelle is astonishing. PolyP’s unique distribution aligns with the localization of proteins targeted by lysine polyphosphorylation, a non-enzymatic post-translational modification driven by polyP.^24^ Initially discovered in the budding yeast *Saccharomyces cerevisiae* and validated by genetic approaches, lysine polyphosphorylation was described in two nucleolar targets in the first instance: NSR1, the yeast homolog of nucleolin, and TOP1 topoisomerase. Subsequent screenings expanded lysine-polyphosphorylation targets to several yeast and mammalian proteins, most of which localize within the nucleolus and are crucial for modulating its physiology.^25^^,^^26^ Interestingly, polyP also interacts with nucleolar proteins in trypanosomatids.^27^

The nucleus organizes genetic material into heterochromatin and transcriptionally active euchromatin. Advances in microscopy have pictured a far more complex organization of the nuclear environment.^28^ Within the nucleus, several nuclear body domains or specialized compartments are now defined. The largest and most famous of these nuclear bodies is the nucleolus, composed of hundreds of transcriptionally active ribosomal DNA (rDNA) repeats.^29^^,^^30^ This subnuclear region is of fundamental importance, as it is where rRNA synthesis, processing, and ribosomal assembly occur. The nucleolus is perhaps the best-documented example of a membraneless organelle generated by liquid-liquid phase separation (LLPS).^31^^,^^32^ Even the different intranucleolar regions are themselves assembled and organized by phase separation phenomena.^33^ Structural organization through phase separation is not confined to the nucleus; many organelles such as the centriole^34^ and the synaptic vesicle,^35^ for example, have been suggested to originate through LLPS. Although the focus has primarily been on RNA-protein complexes driving nucleolar phase separation, it is important to note that polyP, like RNA, is a charged polymer capable of inducing phase separation in positively charged proteins.^36^

Our work, initially aimed at validating reliable methods for extracting and detecting polyP to advance mammalian polyP research, has revealed a selective accumulation of polyP within the nucleolus. We propose that lysine polyphosphorylation of nucleolar proteins targeting polyP within this nuclear body regulates phase separation and induces nucleolar assembly and its associated physiology.

## Results

### Developing a mammalian model to study polyP

To characterize mammalian polyP physiology, it is necessary to develop a reliable experimental model. Ideally, this would be achieved through knocking down/out or overexpressing polyP’s endogenous synthesizing enzyme. Since the mammalian enzymology of polyP synthesis remains unknown,^14^ researchers have resorted to indirect approaches. One method relies on the expression of the *S. cerevisiae* exopolyphosphatase PPX1 to degrade polyP.^37^ This approach presupposes that polyP is present in the first instance. An alternative approach involves the heterologous expression of *Ec*PPK1. When expressed in yeast, *Ec*PPK1 leads to the synthesis of non-vacuole polyP, which is toxic.^38^ PolyP toxicity has also been reported in mammalian cells transiently transfected with *Ec*PPK1.^39^ While transient transfections are easy experiments to perform, the results are not reliable due to the variability of the transfection efficiency. This variability is enhanced by *Ec*PPK1-induced cell toxicity/death, as it is impossible to distinguish poor transfection efficiency from a reduced number of transfected cells between experiments. These considerations prompted us to invest considerable effort into developing an inducible *Ec*PPK1 cell line using Invitrogen’s T-REx system, a tetracycline-regulated expression system that controls *Ec*PPK1 expression via the Tet repressor (TetR) protein (Figure S1). We utilized T-REx-293, derived from HEK-293 (ATCC CRL-1573), already developed to express TetR, and transformed it with an *E. coli* Myc-tagged PPK1 construct (see STAR Methods). We obtained three clonal cell lines, two of which, after tetracycline induction, expressed the Myc-tagged PPK1 protein, inducing cellular accumulation of polyP: TREx-PPK1#1 and TREx-PPK1#3 (Figures S1A and S1B).

### Validation of the TREx-PPK1 lines

**Figure 1:**
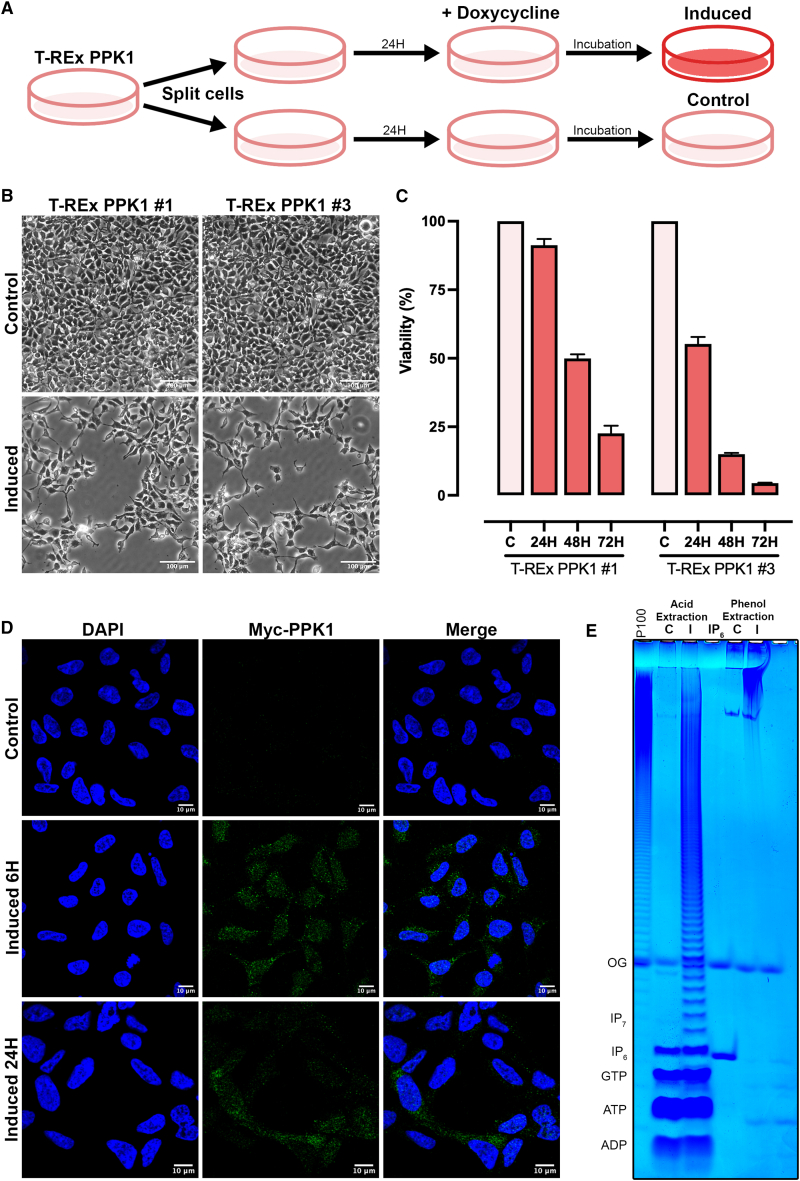
Characterization of TREx-PPK1 cells (A) Schematic workflow for using the doxycycline-inducible TREx-PPK1 lines. 24 h after splitting the cells, the induced group was treated with 1 μg/mL doxycycline. Incubation times varied based on the specific experiment, ranging from 6 to 72 h. (B) Qualitative cell viability assessment of TREx-PPK1 cells using light microscopy after 48 h of induction with doxycycline. Scale bar, 100 μm. (C) Cell viability analysis using the sulforhodamine B assay demonstrates that the viability of induced TREx-PPK1 cells decreases over time. (D) Localization of *Ec*PPK1. Confocal microscopic analysis of the expression of Myc-*Ec*PPK1 after 6 and 24 h doxycycline induction. TREx-PPK1 cells were fixed in 4% paraformaldehyde and permeabilized. Expressed Myc-PPK1 was detected with an anti-Myc monoclonal antibody (green), and the nucleus was stained with DAPI (blue). Scale bar, 10 μm. (E) PAGE analysis of acid/TiO_2_-extracted (10% of extract) and phenol-extracted (10 μg RNA) polyP control (C) and 24 h doxycycline-induced (I) TREx-PPK1 stained with toluidine blue. The migration of the inositol hexakisphosphate (IP_6_) standard was used to define the nature of the bands, following a previously characterized pattern.^40^ The dye Orange G (OG) was used as a migration standard, while polyP_100_ (P100) was used to guide the gel. See also Figure S1.

We employed an experimental protocol as schematized in Figure 1A. Twenty-four hours after splitting the TREx-PPK1 cells, *Ec*PPK1 expression was induced with doxycycline (Doxy), a metabolically stable analog of tetracycline, dissolved in DMEM in one of the sister plates. Both Doxy-induced and control cells were processed in parallel at the designated times. Inducing *Ec*PPK1 expression in clones #1 and #3 was toxic to the cells, causing division arrest and detachment over time (Figure 1B). Quantitative analysis using the sulforhodamine B assay (Figure 1C) revealed that while TREx-PPK1#1 ceased dividing and detached from the plate within 72 h, TREx-PPK1#3 exhibited a stronger toxicity phenotype with faster detachment (48 h). This variation likely reflects the different levels of *Ec*PPK1 expression between the two TREx-PPK1 clonal lines (Figure S1A). We used the TREx-PPK1#1 clone (hereafter labeled TREx-PPK1) for the rest of our analysis, as the relatively mild toxicity phenotype allows us to perform analyses at 24 and 48 h post-induction.

Doxy rapidly induces *Ec*PPK1 expression, detectable by immunohistochemistry within 6 h (Figure 1D). *Ec*PPK1 is found throughout the cell, both in the cytoplasm and the nucleus. We next tested the ability to extract polyP from control and induced cells using two standard procedures: (1) acidic extraction coupled with TiO_2_ enrichment used to improve the recovery of phosphate-rich metabolites^40^ and (2) standard polyP purification through phenol extraction co-purifying RNA/polyP.^41^ The purified extracts were loaded on a 30% PAGE gel, and polyP was visualized with toluidine blue staining (Figure 1E). In line with the polymeric nature of polyP, acidic/TiO_2_ extraction revealed a clear polyP ladder in the induced sample but not in the control. This method also enriches for phosphate-rich metabolites, allowing us to detect nucleotides such as ATP, GTP, and inositol hexakisphosphate, which were the same in control and induced cells (Figure 1E). The analysis of phenol-extracted samples revealed a different pattern. Only the induced extract reveals a large band/smear toward the top of the gel, likely representing high polymeric forms of polyP. To verify this, we treated the phenol extract with the exo- and endopolyphosphatases PPX1 and DDP1, respectively (Figure S1C). Both treatments degraded the smearing band, confirming its nature as polyP. Conversely, the sharp band 4–5 cm from the well remained intact, indicating it is not polyP but likely a small RNA species. Toluidine blue detection of polyP by PAGE is less sensitive than DAPI photobleaching.^42^ The reassessment of our polyP extracts using DAPI photobleaching confirmed the presence of polyP in induced samples but failed to detect any reliable polyP signal in the control extracts (Figure S1D). The phenol extraction method confirmed high polymeric polyP species, suggesting acidic/TiO_2_ extraction, despite showing a pleasing polyP ladder, degrades *Ec*PPK1-synthesized polyP.

### PolyP microscopy detection

**Figure 2:**
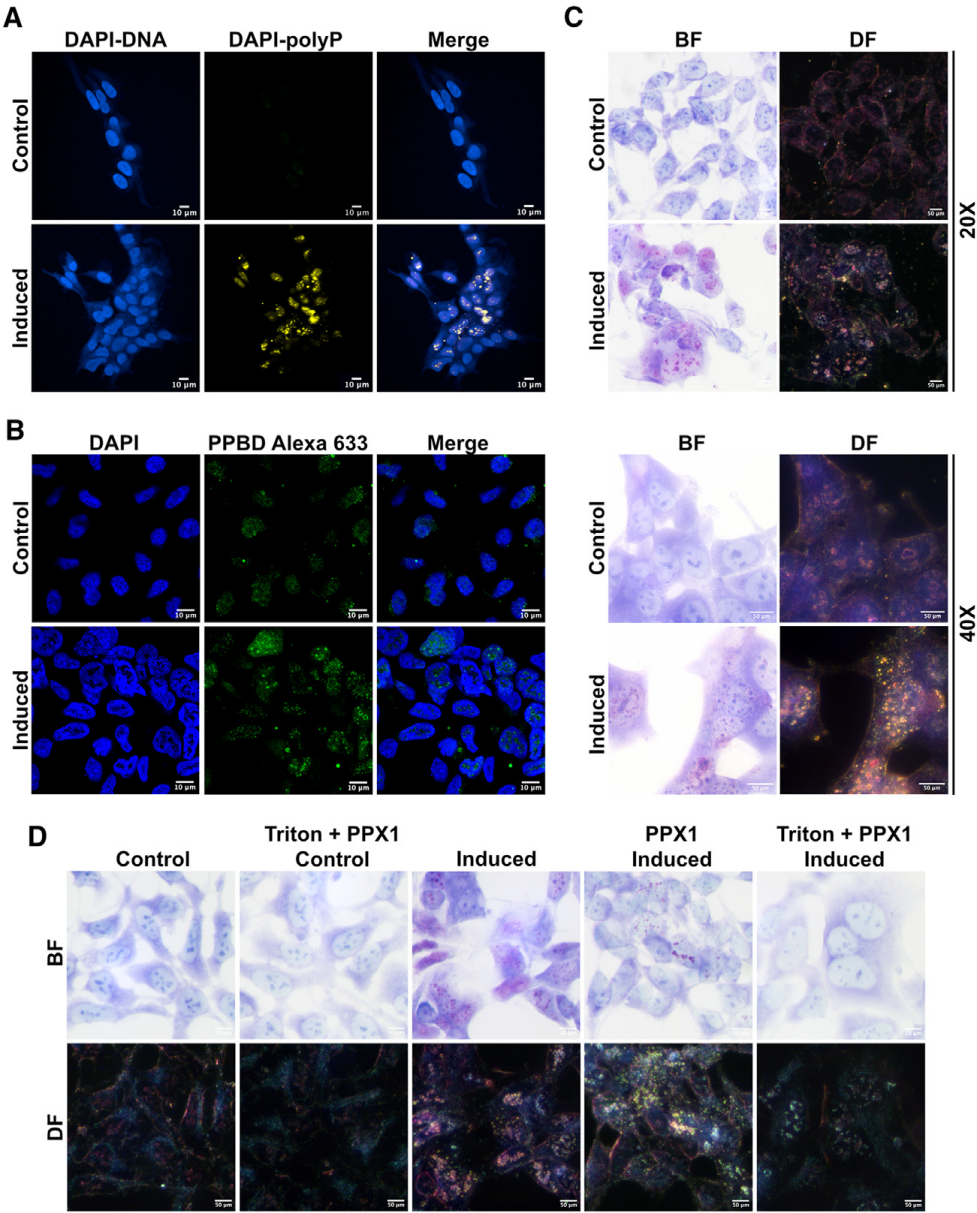
Detection of polyP signal using microscopy (A) Confocal immunofluorescence analysis was performed to detect a polyP-induced DAPI fluorescence shift. Under excitation at a wavelength of 360 nm, the maximum emission wavelength of DAPI-DNA (blue) sits at 460 nm. The red-shifted emission of DAPI-polyP (green) is recorded at wavelengths 475–525 nm under the same excitation. Scale bar, 10 μm. (B) Confocal immunofluorescence analysis was also performed to detect polyP using a polyP-binding domain (PPBD) affinity labeled with Alexa Fluor 633 (green). The nucleus was stained with DAPI (blue). Scale bar, 10 μm. (C) Bright-field (BF) and dark-field (DF) images of TREx-PPK1 cells were captured after toluidine blue staining at 20× and 40× magnification. Scale bar, 50 μm. (D) The effect of polyP degradation on the toluidine blue signal was observed in BF and DF images captured for TREx-PPK1 cells, both treated and untreated with recombinant His-PPX1 for 1 h at 37°C. The group permeabilized with Triton underwent treatment with 0.1% Triton X-100 in PBS for 15 min before PPX1 treatment. Scale bar, 50 μm. See also Figure S2.

One of the methods used to visualize polyP takes advantage of its ability to induce a red shift of DAPI fluorescence.^43^^,^^44^ That said, the ability of DAPI to visualize polyP in cells has been questioned.^19^^,^^20^ DAPI is widely used to visualize the nucleus given that DAPI-DNA has a well-known fluorescence property with an excitation peak at 360 nm and an emission peak at 460 nm.^45^ Conversely, when DAPI interacts with polyP, a shift of DAPI emission excitation at 405–415 nm generates an emission peak of ∼550 nm. Analysis of TREx-PPK1 cells reveals a strong red-shifted DAPI signal in the induced cells, with no signal detected in the control sample (Figure 2A). Despite *Ec*PPK1’s cellular presence, it is surprising to find that most of the polyP-DAPI signal localizes inside the nucleus 48 h post-induction. This analysis takes advantage of the TREx-PPK1 cells as a “positive” control. However, endogenous polyP in non-induced cells failed to be detected using the DAPI red shift in fluorescence. Two additional fluorescence probes, JC-D7 and JC-D8, have been developed to detect polyP.^46^ Compared to DAPI, these probes appear to have higher specificity but lower fluorescence intensity.^46^ We also validated these dyes with TREx-PPK1 cells, but our analysis failed to detect any reliable fluorescence signal in both control and induced cells (Figures S2A and S2B).

Saito et al. developed another method for visualizing polyP, which uses the high affinity of the polyP-binding domain (PPBD) from *E. coli* polyP exopolyphosphatase.^47^ Probing the presence of polyP in induced cells with the PPBD reveals a punctate nuclear staining, consistent with the localization observed in the polyP-DAPI signal (Figure 2B). Importantly, in the control cells, a weaker, predominantly nuclear, and perinuclear polyP signal is also detected, demonstrating that the PPBD might constitute a useful probe to visualize endogenous polyP (Figure 2B). However, only when the polyP synthesizing enzyme is identified and its knockout seen to abolish the PPBD signal can we can be fully confident of its ability to detect endogenous polyP.

Toluidine blue, a basic dye with high affinity for negatively charged groups, is used to stain polyP and inositol phosphates on PAGE (Figure 1E).^48^ We tested its staining ability on control and induced TREx-PPK1 cells. Toluidine blue stains single-stranded RNA better than DNA-protein complexes.^49^ In control cells, the cytosol appeared to be stained blue, with clearer nuclei and visible rRNA-rich nucleoli (Figures 2C and 2D, bright field). Induced cells showed pink/red nuclear-localized spots (Figure 2C, bright field). Toluidine blue retains its blue color with nucleic acid but turns pink/red when bound to polyP,^50^ showing metachromatism (Figure S2C). This property explains the distinct pink/red spots and the absence of the usual blue nucleoli stain in induced cells (Figures 2C and 2D, bright field).

Alongside bright-field microscopy, we examined control and induced TREx-PPK1 cells using dark-field microscopy. In this method, only the light that is scattered (reflected, refracted, or diffracted) by the specimen enters the objective (Figure S2D).^51^ The scattering phenomenon occurs when light encounters particular objects possessing differential density. Dark-field microscopy of TREx-PPK1-induced cells reveals an even more dramatic pink/red punctate nucleus staining, indicating that the polyP that accumulates inside the nucleus forms a dense structure capable of scattering light (Figures 2C and 2D, dark field). In control cells, dark-field microscopy reveals a weak reddish halo defining the nucleolus structures.

To confirm toluidine blue’s specificity for polyP, we treated control and induced TREx-PPK1 cells with PPX1. In permeabilized PPX1-treated induced cells, nuclear pink/red spots disappeared, indicating that these structures are polyP rich (Figure 2D). This treatment allowed visualization of a small blue dot rather than the large nucleoli typically seen in control cells, suggesting that the nucleolar architecture might be altered by polyP (Figure 2D). To check that the toluidine blue data were clone independent, we transiently transfected HeLa and HEK293 cells with *Ec*PPK1. In both cell lines, similar nuclear-localized puncta were observed in bright- and dark-field microscopy (Figure S2E), confirming the findings in induced TREx-PPK1 cells.

### Nucleolar accumulation of polyP

**Figure 3:**
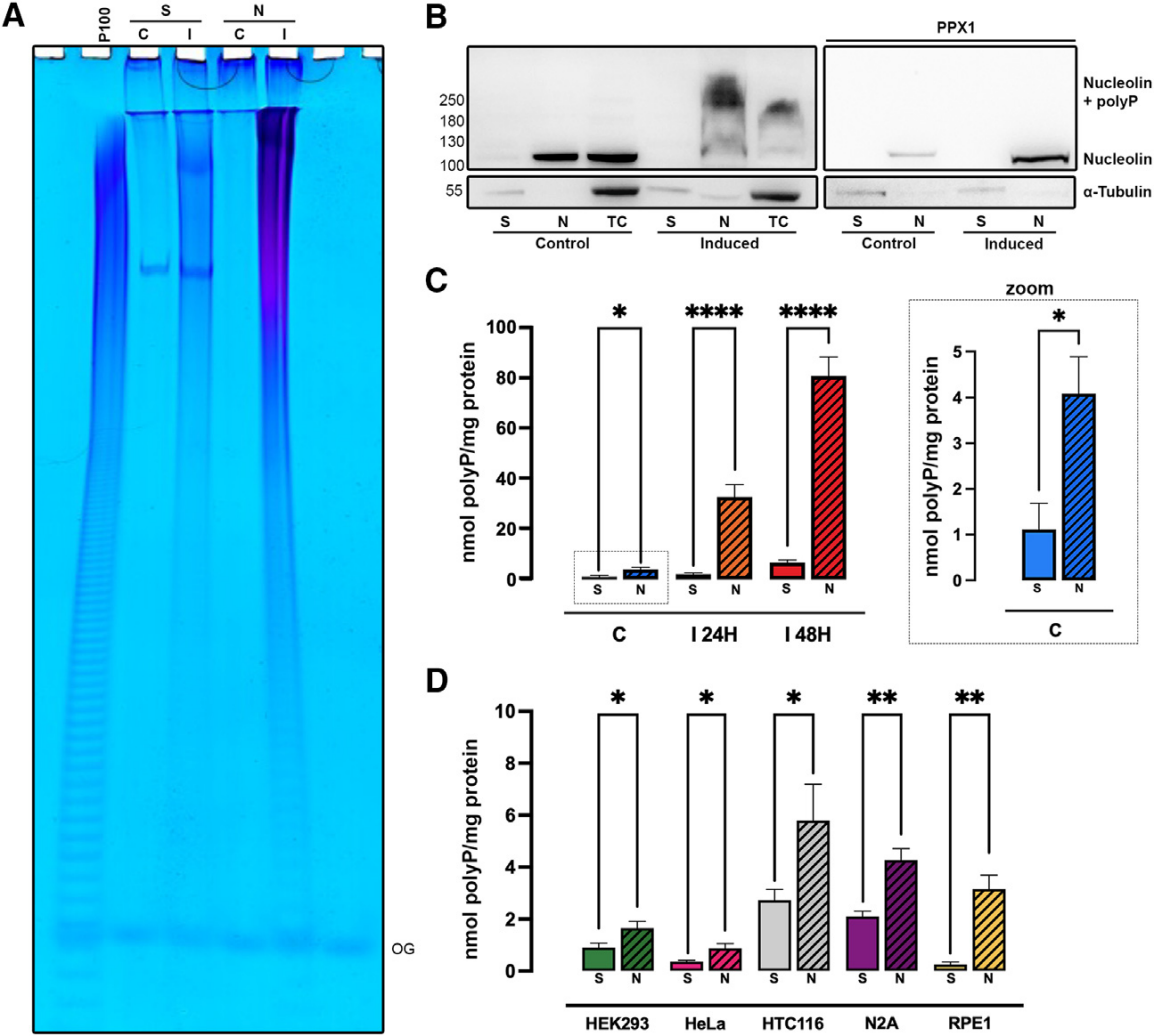
Nucleolar accumulation of polyP (A) Phenol-extracted polyP from the supernatant (S) and nucleoli pellet (N) isolated from TREx-PPK1 control (C) and 48 h induced cells (I) was resolved on a 30% PAGE and stained with toluidine blue. The gel was loaded with half of the supernatant extract and one-fifth of the nucleoli pellet extract. This analysis revealed that virtually all (>95%) of the polyP produced by PPK1 could be recovered in the nucleoli pellet, while the supernatant contained a minimal amount of polyP. OG was used as the migration standard, and P100 was used to guide the PAGE. (B) The distinctive lysine-polyphosphorylation NuPAGE nucleolin mobility shift can be observed in induced cells. Protein extracts from total cells (TCs), nucleoli pellet (N), and supernatant (S) after nuclei isolation from TREx-PPK1 control (C) and induced (I) cells were subjected to western blot analysis with anti-nucleolin and α-tubulin as a control. After degrading polyP with PPX1, the mobility shift disappeared (right). Notably, the cytosolic pool of nucleolin reported in the supernatant (S) of induced cells is absent (left), instead accumulating more evidently in the nucleoli after removing the smeary shift by degrading polyP with PPX1 (right). See also Figure S3. (C) Quantitative polyP determination, expressed as phosphate concentration after PPX1 treatment using the malachite green assay, of supernatant and nucleoli isolated from TREx-PPK1 control (C) and cells induced for 24 h (I24H) and 48 h (I48H) confirms the exclusive accumulation of *Ec*PPK1-synthesized polyP in the nucleoli of induced cells. Importantly, this quantitative assay reveals that in control cells, endogenous polyP accumulates in the nucleoli (zoomed-in box). Results from 3–4 independent experiments were normalized to the protein concentration extracted from sister plates. (D) The accumulation of polyP in the nucleoli is a common characteristic of several mammalian cells. Malachite green quantification of phenol-extracted polyP from the supernatant (S) and nucleoli pellet (N) of HEK293, HeLa, HCT116, N2A, and RPE1 cells. Data are represented as mean ± SEM, showing levels of significance at ∗*p* < 0.05, ∗∗*p* < 0.01, and ∗∗∗∗*p* < 0.001.

The unexpected nuclear localization of polyP in induced TREx-PPK1 and the apparent nucleolus disorganization prompted us to purify this subnuclear organelle using a sucrose cushion.^52^ After nucleolus isolation, we phenol extracted the resuspended pellet and the supernatant representing the other cellular components. In accordance with the microscopy analysis (Figures 2A–2C), in induced cells, virtually all polyP was recovered from the nucleoli pellet (Figure 3A). Western blot analysis of nucleolin validated the quality of our nucleoli purification (Figure 3B). In the control cells, nucleolin was detected in the nucleoli fraction, and a weak band was also present in the supernatant, confirming that nucleolin shuttles between the cytosol and nucleolus, with 5%–10% residing in the cytosol.^53^ Conversely, in the induced cells, nucleolin was found to only localize to the nucleolus. Additionally, in induced cells, nucleolin showed a dramatic upward mobility shift that disappears with PPX1 treatment, a known characteristic of protein polyphosphorylation (Figure 3B, right).^24^ This mobility shift supports previously published literature^26^ and is further observed in the *Ec*PPK1 transient transfected HEK293 cells (Figure S3A).

Quantitative polyP measurement involves enzymatic degradation with PPX1 and subsequent quantification of the released phosphate using a malachite green assay. Quantitative polyP analysis in nucleolar and supernatant fractions of the TREx-PPK1 cell line revealed a 20-fold increase in polyP in the induced cells when compared to control (Figure 3C). Interestingly, this analysis revealed that in control cells, most of the endogenous synthesized polyP is found in the nucleolus (Figure 3C, zoomed-in box). This prompted us to investigate if endogenous polyP nucleolar localization was a universal characteristic in mammalian cells. We purified the nucleoli from five different mammalian cell lines and quantitatively measured polyP levels. In all cell lines assayed, we recorded a statistically significant increase between the amount of polyP present in the nucleoli pellet and that recovered from the supernatant (Figure 3D). Therefore, the unexpected and selective accumulation of polyP in the nucleoli of induced TREx-PPK1 could be interpreted as channeling polyP to where its natural physiological function takes place.

### PolyP modifies nucleolar organization

**Figure 4:**
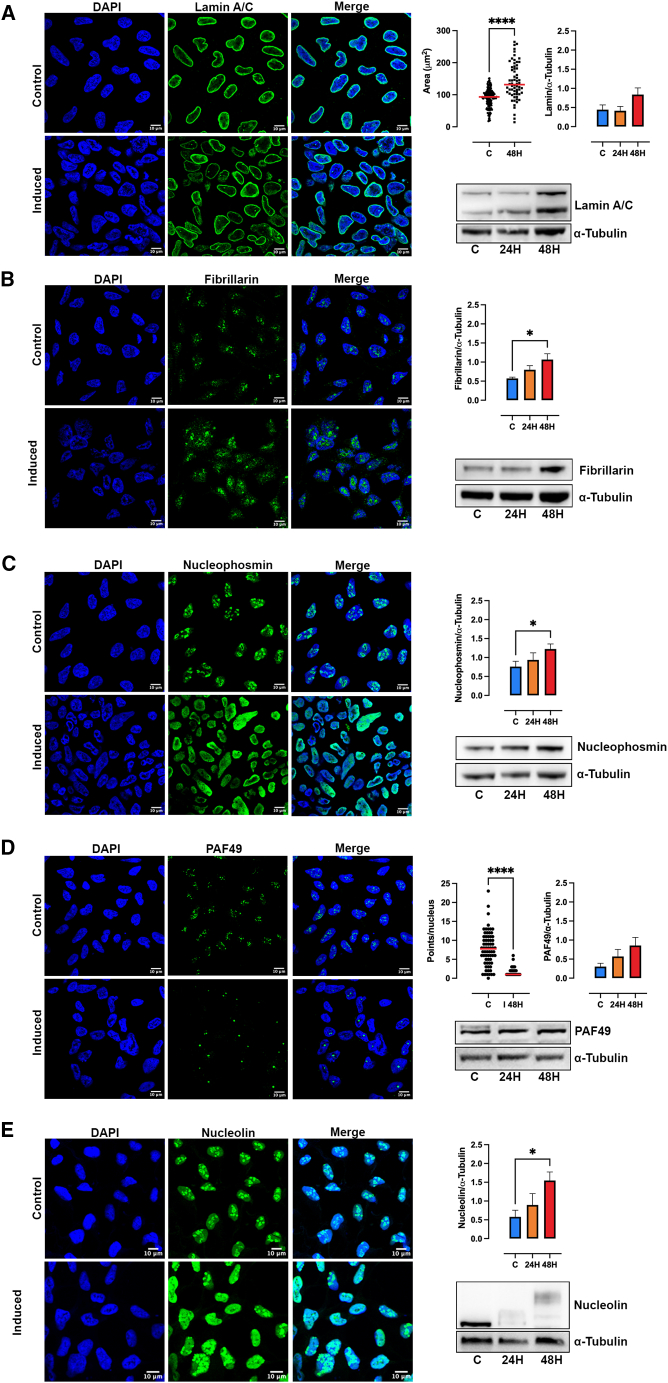
polyP induced alterations in nucleolar organization TREx-PPK1 control and cells induced for 48 h were fixed and stained with specific antibodies for lamin A/C (A), fibrillarin (B), nucleophosmin (C), PAF49 (D), and nucleolin (E). Scale bar, 10 μm. The nucleus was stained with DAPI (blue), and the different nucleolar markers are visualized in green. Each marker corresponds to its respective western blot analysis for control and induction after 24 and 48 h with doxycycline, normalized to α-tubulin. The nucleus area was determined using ImageJ on DAPI staining (A). The analysis for PAF49 spots per nucleus was also performed using ImageJ (D). Quantification was carried out based on at least three independent experiments. Data are represented as mean ± SEM, and the significance level was set at ∗*p* < 0.05 and ∗∗∗∗*p* < 0.001.

The almost exclusive nucleolar localization of polyP prompted us to characterize the nuclear/nucleolar organization. To study general nuclear morphology, we assessed laminin, a matrix protein on the inner nuclear membrane crucial for chromatin organization.^54^ Confocal imaging of laminin A/C reveals that the nuclei in induced cells remain intact but are significantly enlarged, without a change in laminin A/C expression relative to tubulin (Figure 4A).

The nucleolus is subdivided into structurally and functionally diverse subregions: the fibrillar center (FC), the dense fibrillar component (DFC), and the granular component (GC) (for review, see Baserga et al.^29^ and Lafontaine et al.^33^). To study alterations in nucleolar organization, we monitored fibrillarin, a major DFC protein, and nucleophosmin, a GC protein. Confocal imaging of fibrillarin in control cells revealed a typical nuclear punctate pattern, defining the position of the nucleoli (Figure 4B). This pattern is conserved in induced cells; however, part of the fibrillarin signal was found to be diffused around the puncta and with an increased signal intensity. In line with the confocal results, western blot analysis revealed an increase in fibrillarin protein levels in induced cells (Figure 4B). Confocal imaging of nucleophosmin showed the characteristic punctate signal in control cells. The presence of polyP in induced cells caused a striking redistribution of nucleophosmin throughout the nucleoplasm, with protein level increases confirmed by western blot analysis (Figure 4C). The FC was also tracked using RNA polymerase I subunit PAF49. While several PAF49 loci could be seen in control cells, very few, often just one, PAF49 puncta were observed in induced cells (Figure 4D). Finally, we monitored nucleolin, the most abundant non-ribosomal protein present in the nucleolus. As expected, nucleolin was distributed in nucleolar puncta in control cells; however, in induced cells, the presence of polyP led to the redistribution of nucleolin throughout the nucleoplasm (Figure 4E), a pattern similar to that observed for nucleophosmin (Figure 4C). In a few induced cells, a spotty pattern could still be observed. Western blot analysis revealed a nucleolin mobility shift observable at 24 h post-induction, which became more dramatic at 48 h (Figure 4E). With the observation that polyP excess alters the nucleolar architecture in induced cells, we analyzed one of the primary functions of this membraneless organelle: rRNA transcription and processing.

### Excess of polyP altered nuclear RNA processing

**Figure 5:**
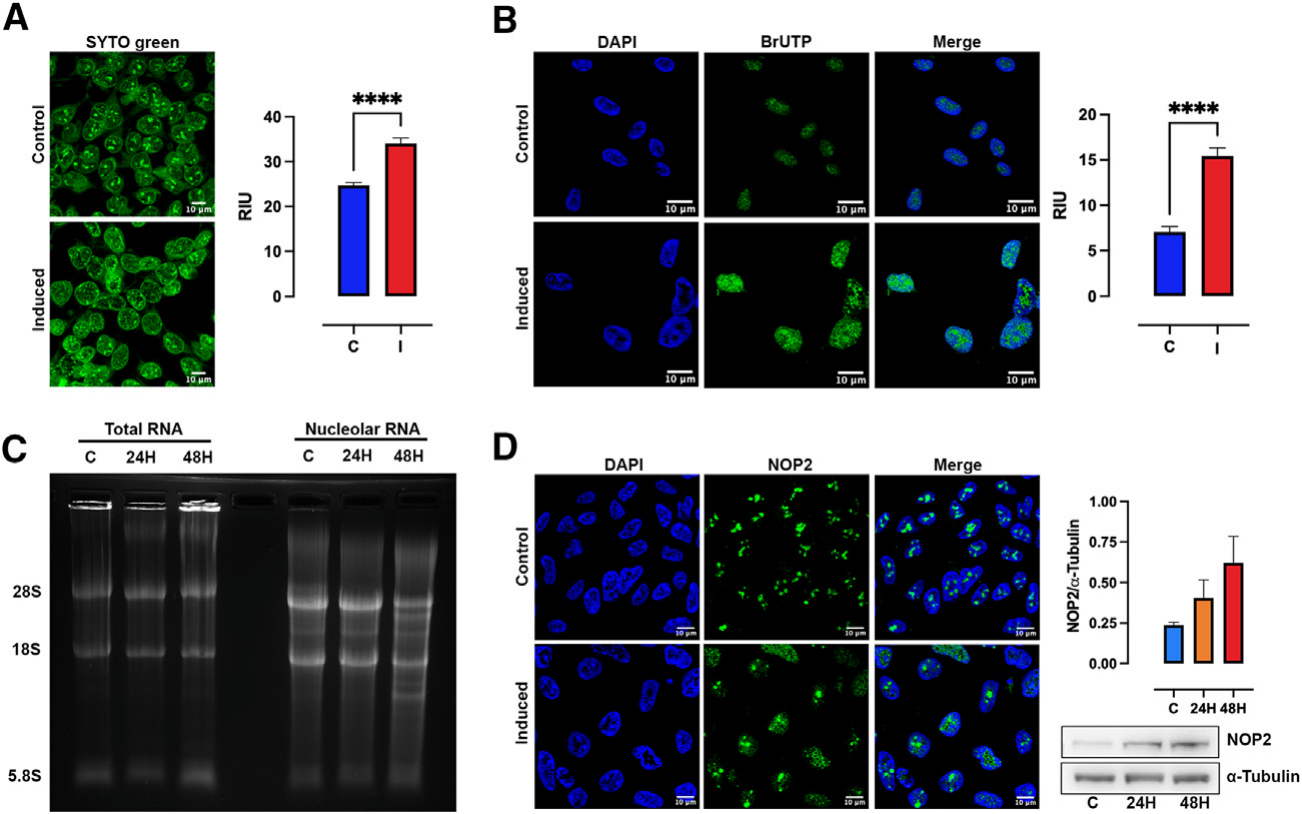
Changes in rRNA transcription and processing by polyP (A) Immunofluorescence staining for SYTO RNASelect Green. The analysis of its net fluorescence intensity quantification was conducted on TREx-PPK1 cells in control conditions and after 48 h of induction (I). Scale bar, 10 μm. (B) Run-on transcription assay and its net fluorescence intensity quantification were performed. Newly synthesized RNA was visualized through the BrUTP signal (green), and the nucleus was stained with DAPI (blue). Scale bar, 10 μm. Quantification was carried out based on at least three independent experiments. Data are represented as mean ± SEM, and the significance level was set at ∗∗∗∗*p* < 0.001. (C) Defects in rRNA processing induced by polyP. Gel electrophoresis of phenol-extracted RNA from TCs and isolated nucleoli from TREx-PPK1 control (C) and after 24 (24H) and 48 h (48H) of induction was conducted. (D) Immunofluorescence staining for NOP2 (green) and its respective western blot analysis for control (C) and after 24 (24H) and 48 h (48H) of induction, normalized to α-tubulin, were carried out. The nucleus was stained with DAPI (blue). Scale bar, 10 μm.

We used the specific RNA dye SYTO RNASelect green to monitor general transcriptional output in control and induced cells. SYTO RNASelect green is used to study nucleoli physiology.^55^ In control cells, the rRNA transcription occurring within the nucleoli can be observed, in addition to perinuclear staining (Figure 5A). Induced cells showed a diffuse, more intense nuclear signal, suggesting an enhanced transcriptional output (Figure 5A). To test this hypothesis, we performed a transcription run-on assay with BrUTP for 30 min. Both control and induced cells displayed similar BrUTP incorporation patterns in the nucleus, with higher incorporation in areas corresponding to rRNA-rich nucleoli (Figure 5B). Importantly, while the pattern of the BrUTP signal appeared unaltered between control and induced cells, the intensity of signal increased 2-fold in induced cells, suggesting that excess polyP enhances transcriptional output (Figure 5B).

To verify if polyP affects rRNA processing, we phenol extracted RNA from control induced cells (total RNA) and previously purified nucleoli (nucleolar RNA). In mammalian cells, rRNAs represent ∼80% of all RNA, making it easy to detect 28S, 18S, and 5S RNA signals on an agarose gel. Total RNA extract analyses revealed no change in rRNA levels or processing between control and induced cells (Figure 5C), as expected due to the long half-life of mammalian rRNA.^56^ However, nucleolar rRNA analysis revealed differences between control and induced cells (after 48 h of induction), with the presence of additional bands, suggestive of altered rRNA processing (Figure 5C). A key rRNA processing protein is NOP2/NSUN1, which methylates cytosines at specific pre-rRNA positions, driving pre-rRNA folding and subsequent processing.^57^ Additionally, NOP2/NSUN1 has been proposed to non-catalytically regulate pre-rRNA processing by recruiting additional factors.^58^ Confocal NOP2/NSUN1 imaging in control cells displayed a characteristic nucleolar foci pattern compatible with its nucleolar location. However, in induced cells, NOP2/NSUN1 revealed a diffused signal, supporting the observation that polyP excess alters rRNA processing. Western blot analysis showed a tendency for NOP2/NSUN1 protein expression to increase with induction time; however, this was not statistically significant (Figure 5D).

### PolyP induces nucleoli condensation

**Figure 6:**
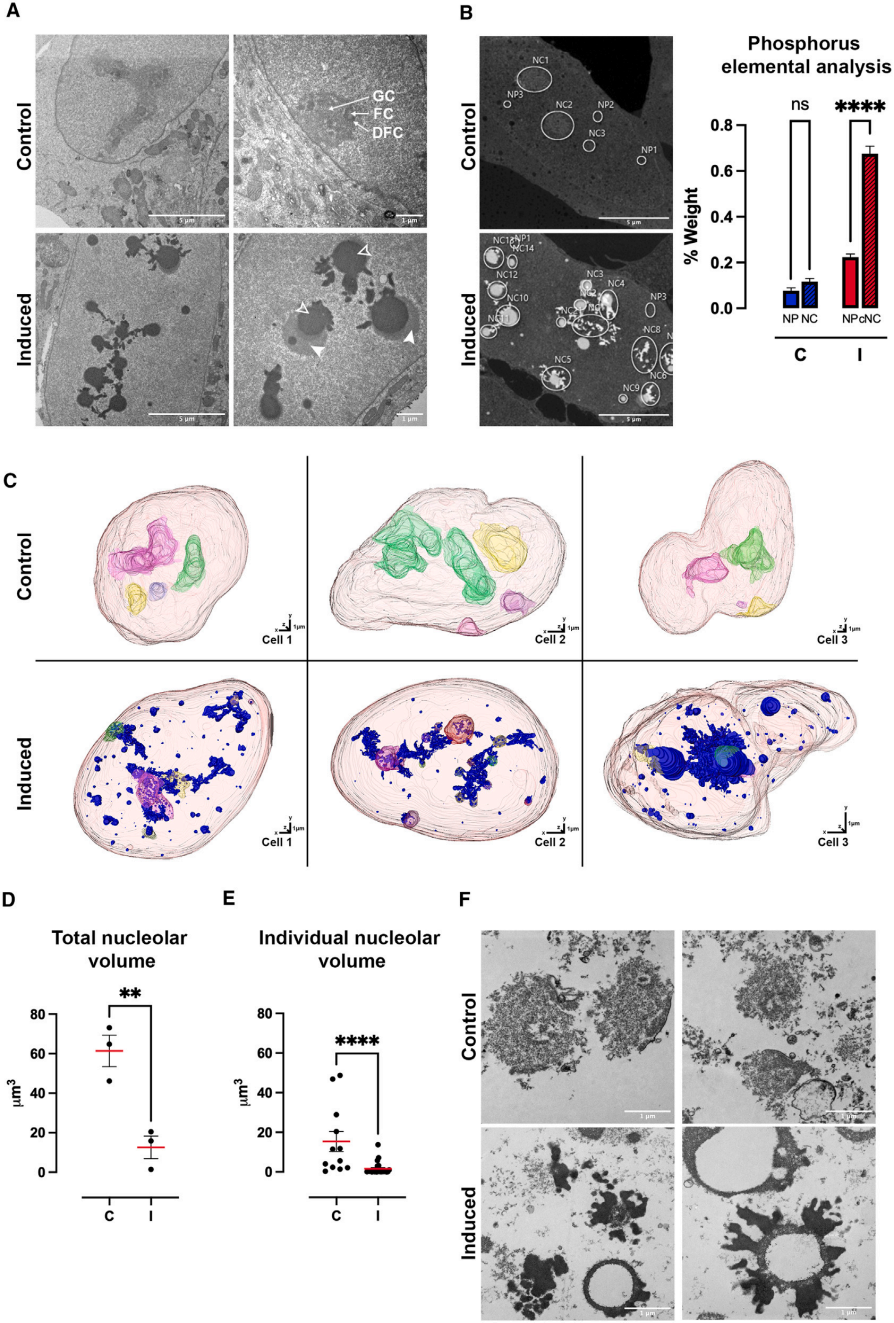
polyP induces nucleoli condensation (A) Transmission electron microscopy of unstained ultrathin sections (70 nm) of TREx-PPK1 cells from control and induced cells (after 48 h of induction with doxycycline). Filled or open arrowheads indicate regions with medium or extreme increases in electron density, respectively. Scale bar, 1 or 5 μm. (B) Elemental analysis using energy-dispersive X-ray spectroscopy (EDS). Scanning electron microscopy (SEM) from control (C) and after 48 h of induction (I). Phosphorus analysis from spectra collected from nucleoplasm (NP), nucleoli (NC), and nucleoli + electron-dense structures (cNC). The percentage weight of each element per subdomain was averaged for each cell. Scale bar, 5 μm. (C) Three-dimensional (3D) reconstruction of array tomography SEM images of the nucleus from control and after 48 h induction with doxycycline of TREx-PPK1 cells. Scale bar, 1 μm. (D and E) Quantification of total nucleolar volume (D) and individual nucleolar volume (E) from 3D reconstruction of TREx-PPK1 cells in (C). (F) Transmission electron microscopy of lead citrate-stained ultrathin sections of embedded isolated nucleoli from TREx-PPK1 cells, both control and after 48 h of induction with doxycycline. Quantifications were carried out based on at least three experiments. Data are represented as mean ± SEM, and the significance level was set at ∗∗*p* < 0.01 and ∗∗∗∗*p* < 0.001; ns, non-significant. Scale bar, 1 μm. See also Figure S4.

Given that polyP synthesis in induced TREx-PPK1 cells rearranges many nucleolar markers, co-localization experiments to elucidate its internal organization may be misleading. It is also possible that in induced TREx-PPK1 cells, the nucleoli become muddled within the nucleoplasm. To confirm nucleoli presence in induced cells, we used transmission electron microscopy (TEM) to visualize them. The nucleoli are easily recognizable by TEM as differentially dense structures within the nucleoplasm.^59^ In control cells, nucleoli were characteristically large structures, more electron dense than the surrounding nucleoplasm, with well-defined electron density regions defined as GC, DFC, and FC in Figure 6A^29^^,^^33^ Consistent with previous analyses,^59^ in TEM images of unstained ultrathin sections, the nucleoli of control cells appeared in various shapes, from roughly roundish to elongated with amorphous boundaries (Figure 6A). In induced cells, while the nucleus is well defined, the intranuclear structures present altered morphologies. We observed several foci with smooth, well-defined boundaries, often round with emanating tubular or round protrusions, displaying minimal texture with either a medium (Figure 6A, filled arrowheads) or extremely increased (Figure 6A, open arrowheads) electron density. Extremely dense structures appear to be associated with structures of medium increased electron density, suggesting that in the presence of polyP, nucleoli are compacted into circular structures of homogeneous appearance (Figure 6A).

We hypothesize that the increased electron density of the dark foci (open arrowheads) in induced cells could be generated by polyP’s ability to interact with cations in cells or with heavy metals during TEM sample preparation. Further investigation through energy-dispersive X-ray spectroscopy (EDS) scanning electron microscopy (SEM) revealed that the electron-dense spots contain significantly more phosphorous than any other nuclear region analyzed (Figure 6B). Consequently, we now identify these structures as polyp-rich domains.

Our two-dimensional (2D) TEM analysis revealed that polyP reorganizes nucleoli into compact circular structures. To delve deeper into this polyP-induced reorganization and explore the relationship between electron-dense and polyP-rich domains, we performed array tomography imaging by SEM followed by 3D reconstruction. In control nuclei, we identified four distinct nucleoli—two small and two large—that formed elongated organelles inside the nucleoplasm (Figure 6C; Videos S1, S2, and S3). Notably, all four nucleoli appear to make contact with the nuclear envelope. In induced cells, the nucleoli defined by the higher-density region are condensed into a couple of roughly spherical structures, resembling the size of the two smaller nucleoli present in control cells. Additionally, numerous small spheres of higher density, possibly fragmented nucleoli or other nuclear condensates such as Cajal body and speckles,^60^ could be identified (Figure 6C; Videos S4, S5, and S6). The nucleoli spheres were embedded within a 3D network of extremely dense structures, the polyP-rich domains (Figure 6C; Videos S4, S5, and S6, blue). The number of small significantly dense spheres likely represents an underestimation since the abundant signal of the polyP-rich regions could mask the presence of few small dense spheres. Interestingly, in induced cells, the vast majority of the spherical nucleoli or the smaller spheres do not make contact with the nuclear envelope. The quantification of the total nucleolar volume (Figure 6D) revealed that polyP caused an over two-third reduction in the total nucleolar volume (Figure 6E).

Video S1. Array tomography imaging by SEM followed by 3D reconstruction of control nucleus 1, related to Figure 6C

Video S2. Array tomography imaging by SEM followed by 3D reconstruction of control nucleus 2, related to Figure 6C

Video S3. Array tomography imaging by SEM followed by 3D reconstruction of control nucleus 3, related to Figure 6C

Video S4. Array tomography imaging by SEM followed by 3D reconstruction of induced nucleus 1, related to Figure 6C

Video S5. Array tomography imaging by SEM followed by 3D reconstruction of induced nucleus 2, related to Figure 6C

Video S6. Array tomography imaging by SEM followed by 3D reconstruction of induced nucleus 3, related to Figure 6C

The formation of compacted nucleoli with sharp boundaries of even appearance prompted us to test if the cytosolic membranelles organelle organization regulated by LLPS is affected by polyP. We analyzed two structures, the centriole architecture, which has been proposed to be organized by phase separation phenomena,^34^ and the processing bodies (P-bodies), given that their organization is LLPS dependent and the importance of these ribonucleoprotein granules in mRNA processing.^61^ The analysis of pericentrin, the integral component of the centriole, did not reveal an alteration in centriole numbers or location between control and induced cells (Figure S4A). Similarly, the analysis of the P-body RNA helicase DDX6^62^ revealed no change in number or distribution of P-bodies between control and induced cells (Figure S4B). These findings indicate that it is not the ubiquitously distributed *Ec*PPK1 protein (Figure 1D), and thus polyP synthesis per se, that induces nucleolar condensation but rather the selective accumulation of polyP within this subnuclear compartment.

Finally, to further confirm the identity of these structures as nucleoli, we examined biochemically purified nucleoli using TEM (Figure 6D). Nucleoli purified from control cells exhibited a structure consistent with previous published analyses,^52^ displaying granularity akin to that observed within intact cells (Figure 6A). In contrast, nucleoli purified from induced cells retained their significant and extreme electron density nature, with few purified nucleoli displaying the distinctive significant and extremely dense elements surrounding an empty space. However, it is also possible that the sample preparation process might have impacted the morphology of these purified structures, and further studies are needed to better characterize this phenomenon. Notwithstanding this, the data support the identification of these structures as nucleoli condensates associated with the extremely dense polyP-rich domains as revealed by TEM and EDS analysis (Figures 6A and 6B) and further validate the biochemical studies presented in Figure 3, demonstrating the selective association of polyP with the nucleolus.

### PolyP alters nucleoli LLPS organization

**Figure 7:**
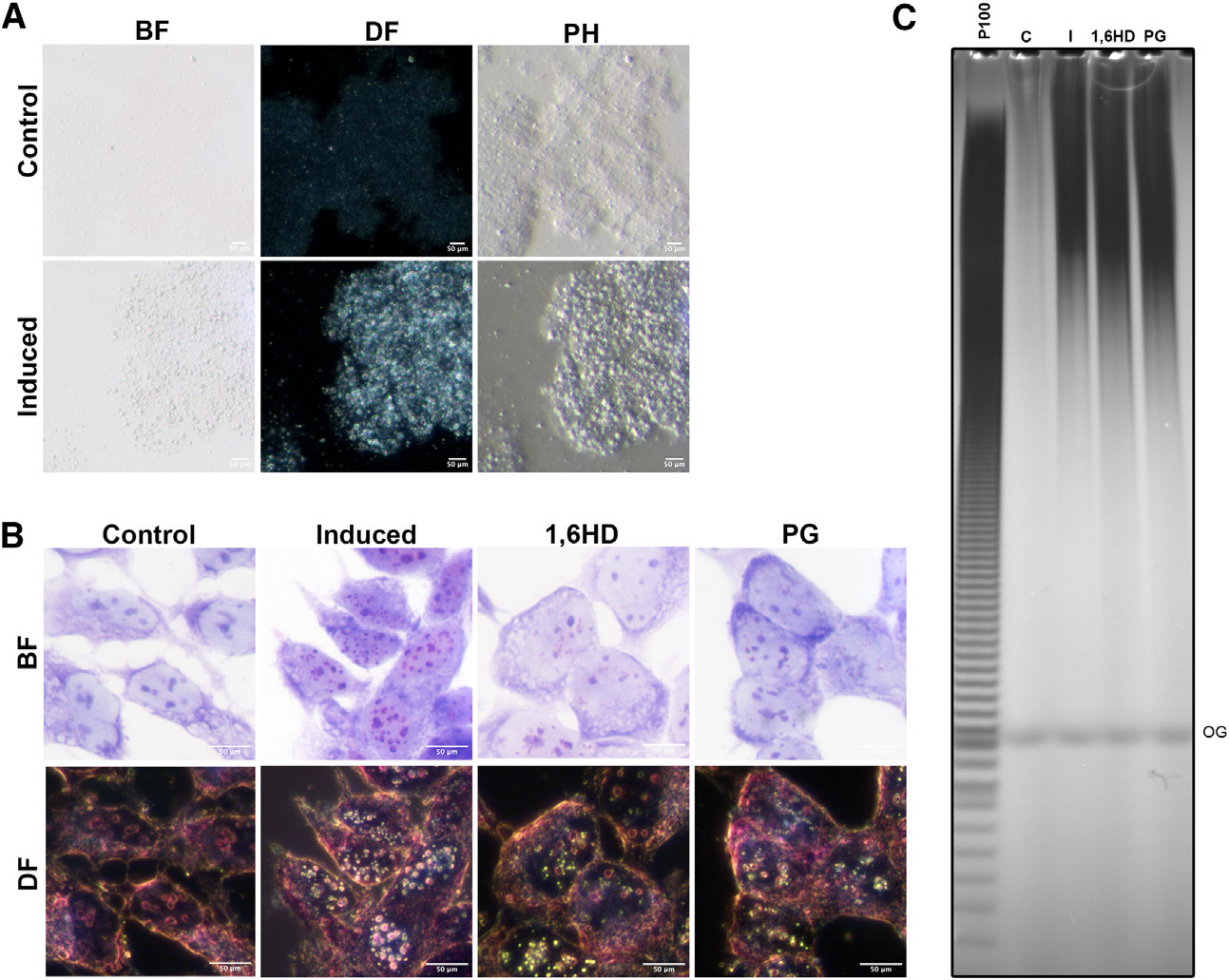
polyP alters the LLPS status of the nucleolus (A) Light microscopy using BF, DF, and phase contrast (PH) were used to observe the isolated nucleoli of TREx-PPK1 cells from control and after 48 h of induction with doxycycline. Scale bar, 50 μm. (B) To disrupt polyP-induced nucleolar condensates, aliphatic alcohols 1,6-hexanediol (1,6HD) and propylene glycol (PG) were utilized. BF and DF images show control and induced (48 h) TREx-PPK1 cells treated with 1,6HD and PG for 60 min before fixation and toluidine blue staining. Scale bar, 50 μm. (C) Phenol extracts (10%) from a parallel experiment were resolved on a 30% PAGE and stained with DAPI. See also Figure S5.

The nucleolus, a prototypical non-membranous organelle, is assembled and organized through LLPS.^31^ Electron microscopy analysis, revealing condensed nucleoli of even appearance with smooth boundaries, suggests that polyP alters nucleolar architecture by influencing LLPS and inducing hyper-condensation. This hypothesis is supported by two observations. First, in the presence of polyP (induced cells), purified nucleoli appear compacted, which is evident not only by TEM but also under optical microscopy (Figure 7A). Nucleoli purified from control cells exhibit a degree of granularity consistent with their already dense nature. In the presence of polyP, nucleoli purified from induced cells show enhanced granularity, which can already be seen with poor contrast techniques like bright-field microscopy. Improved contrast techniques, such as dark field or phase contrast, reveal that polyP increases coarseness and therefore density (Figure 7A). Secondly, recent literature indicates that polyP induces LLPS of positively charged proteins^36^ and can contribute to the formation of phase-separated condensates in bacterial chromatin structure.^63^^,^^64^ Conventional LLPS is typically driven by the charged nature of nucleic acids and their association with DNA- or RNA-specific binding proteins. Highly charged polyP might use its ability to interact with positively charged protein domains^36^ or coordinate cations and induce LLPS. Indeed, simple incubation of polyP with calcium or magnesium can induce phase separation, leading to the formation of round vesicle-like structures, the size of which is dependent on the cations and pH (Figures S5A and S5B).

To investigate whether the nucleoli compaction in induced cells (Figures 4, 5, and 6) is driven by polyP induction of LLPS condensates, we relied on the hydrophobic and hydroxylated nature of propylene glycol (PG) and the aliphatic alcohol 1,6-hexanediol (1,6HD) to disrupt LLPS condensates.^65^^,^^66^ Dark-field microscopy of induced cells stained with toluidine blue reveals that treatment with either PG or 1,6HD results in a near dissolution of the polyP-induced bright pink spots (Figure 7B). Bright-field microscopy revealed a similar pattern: a reorganization of the pink polyP signal, with the nucleoli becoming identifiable due to their blue rRNA staining. A faint pink stain is seen throughout the nucleoplasm, indicating that PG and 1,6HD are merely disrupting polyP-induced LLPS and not breaking down polyP. Indeed, PAGE analysis of extracted polyP demonstrates that these compounds do not degrade polyP, as similar amounts of polyP could be recovered from untreated and PG- and 1,6HD-treated cells (Figure 7C). This result demonstrates that polyP hyper-condensates this organelle by exacerbating its underlying LLPS phenomena.

## Discussion

The developed TREx-PPK1 cellular model has enabled us to address previously debated issues, such as the ability to extract polyP from mammalian cells and visualize it on PAGE. This is only possible with induced TREx-PPK1 cells but not with control non-induced cells, even after phospho-metabolomic enrichment with TiO_2_. This result corroborates previous work that failed to extract polyP from HCT116 cells even after ^32^P-orthophosphate radiolabeling .^21^ The reported ability to detect mammalian polyP by PAGE^67^ could be attributed to the use of RNases (ribonucleases) during sample preparation, which produces small RNA fragments that generate a smeary appearance that could be confused as polyP. Since RNA migrates to the top of the gel, RNase treatment is not necessary to distinguish RNA from polyP (Figure 1E). Moreover, the metachromatic reaction of toluidine blue differentiates bluish RNA from reddish polyP (Figure S2C). Using non-denaturing gels without urea, which polyP does not require due to its lack of a secondary structure, allows higher acrylamide concentrations that enhance the visibility of polyP’s distinctive laddering pattern (Figure 1E).^48^

The valuable role of TREx-PPK1 as a positive control for validating polyP analytical technologies prompted us to develop new methods for studying polyP. We explored using toluidine blue to visualize polyP in induced TREx-PPK1 cells. The metachromasia of toluidine blue-polyP complexes in induced cells revealed a reddish, often spotted staining of polyP, distinct from the RNA blue staining. While the nuclear localization of polyP in induced TREx-PPK1 cells was observed using DAPI and PPBD analysis, toluidine blue staining provided an initial indication of polyp-induced nucleolar disorganization. The large blue spots of rRNA that define the nucleolus in control cells appear significantly smaller, often dotted, co-localizing with the reddish signal of polyP. The stunning images obtained through dark-field microscopy, which generates images from scattered light, hint to the dense nature of reddish polyP aggregates.

The selective localization of polyP targeting the nucleolus was surprising given that Myc-*Ec*PPK1 is localized throughout the cell. However, this exclusive localization closely matches the distribution of lysine-polyphosphorylated target proteins.^24^^,^^26^^,^^68^ Polyphosphorylation occurs at polyacidic serine (S) and lysine-rich (PASK) amino acid clusters. Genome-wide screening to identify PASK proteins revealed that the vast majority of polyP targets are nucleolar proteins, which are crucial for regulating its physiology and ribosome biogenesis.^26^ Therefore, it is logical to assume that lysine polyphosphorylation of nucleolar proteins plays a pivotal role in the distinct polyP localization observed in induced TREx-PPK1 cells. The inability to detect the cytosolic pool of nucleolin in induced cells suggests that polyphosphorylation drives nucleolar translocation. This could explain how polyP synthesized in the cytosol accumulates within the nucleus. As protein phosphorylation is a prerequisite for the nuclear import of numerous cargos,^69^^,^^70^ polyphosphorylation of nucleolar PASK-containing proteins could either drive or contribute to their nuclear translocation.

The nucleolus is rich in proteins that are substantially phosphorylated, such as nucleophosmin, nucleolar and coiled-body phosphoprotein 1 (NOLC1 or NOP140), and nucleolin.^32^^,^^71^^,^^72^ Many of these nucleolar proteins, such as NOPP140, undergo serine pyrophosphorylation, a post-translational protein modification driven by inositol pyrophosphate.^73^^,^^74^ The extensive addition of phosphate groups imposes a negative charge to these peptides. Nucleolar proteins containing the PASK domain, when covalently bound to polyP chains,^68^ introduce a significantly higher number of phosphate groups. What could be the physiological role of creating a phosphate-rich nucleolar environment? This phosphate-rich environment might play a physiological role by enhancing hydrogen bonding with water molecules,^75^^,^^76^ thus creating a substantial hydration shell around proteins. This hydration facilitates a specific biochemical environment,^75^ potentially promoting the condensed phase necessary for the multilayered architecture of the nucleolus, constructed through multiple LLPS elements.

The nucleolus comprises three distinguishable layers: the FC surrounded by the DFC and the GC, which embeds both the FC and DFC structures. These layers are maintained by distinct LLPS phenomena involving diverse complex biomolecular condensates.^31^^,^^33^ LLPS is not exclusive to the nucleolus; other nuclear regions, like nuclear speckles and spliceosomes,^60^ also organize into discrete phases known as LLPS droplet organelles. Our 3D EM reconstruction of polyP distribution in the induced TREx-PPK1 nucleus reveals super-condensed nucleoli, as well as many other smaller condensed structures. These smaller condensates, potentially fragmented DFC regions, are not labeled by the GC marker nucleophosmin or the FC marker PAF49. Alternatively, polyP might influence LLPS in other nuclear bodies, such as nuclear speckles and/or spliceosomes, a hypothesis requiring experimental validation in future studies.

The observed hyper-condensation of the nucleolus aligns with the biophysical properties of polyP, which organize its surroundings by coordinating water and cations (Figure S5). These rational interpretations of unexpected phenotypes emphasize the importance of an unbiased scientific approach. The unexpected discovery that polyP accumulates in the nucleolus should drive further efforts to identify the mammalian enzyme or machinery responsible for polyP synthesis. Is the enzyme that synthesizes polyP localized within the nucleus/nucleolus? This is a reasonable assumption, considering that the other two negatively charged biological polymers, DNA and RNA, are synthesized within the nucleus. The challenging quest to identify the mammalian enzyme responsible for polyP synthesis requires an understanding of the nature of polyP in mammalian cells. Mammals have lower levels of polyP compared to bacteria or yeast,^77^ but they are also likely to possess short polymeric forms of polyP, which are poorly stained by toluidine blue and DAPI,^78^ explaining our inability to visualize them by PAGE. The mammalian polyP endopolyphosphatase was identified as Nudt3,^79^ a member of the NUDIX family previously characterized as a diadenosine polyphosphatase.^80^ For example, diadenosine hexaphosphate (Ap6A) contains a chain of six phosphates with adenosines at both ends. Could the diadenosine polyphosphates represent the short-chain polyP present in mammalian cells? This possibility should encourage further research in this direction.

### Limitations of the study

The heterologous synthesis of polyP has allowed us to uncover its selective accumulation into the nucleolus. While endogenously synthesized polyP also accumulates within this organelle, the lack of knowledge on its synthetic enzyme prevents the assessment of polyP function using a more physiological setting. While the observed nucleolar phenotype is compatible with lysine polyphosphorylation of PASK-containing nucleolar proteins, future work will focus on identifying the exact polyP partner(s) controlling LLPS phenomena. The new technology described here has limitations too. Toluidine blue staining has given the initial hint of polyP nucleolus accumulation and its condensation. However, the current experimental approach is not compatible with simultaneous confocal analysis. Further development of this approach is also required to implement this simple staining for the visualization of endogenously synthesized polyP.

## STAR★Methods

### Key resources table

**Table:** 

REAGENT or RESOURCE	SOURCE	IDENTIFIER
**Antibodies**		

α-tubulin	Biolegend	Cat# 625902; RRID: AB_493416
4′,6-Diamidino-2-Phenylindole (DAPI)	Sigma	Cat# D9542
Alexa Fluor 488	Invitrogen	Cat# A21202; RRID: AB_141607
Alexa Fluor 633	Invitrogen	Cat# A21052; RRID: AB_2535719
Anti-Xpress	Invitrogen	Cat# R910-25; RRID: AB_2556552
DDX6	Invitrogen	Cat# PA5-27786; RRID: AB_2545262
Fibrillarin	Santa Cruz Biotechnology	Cat# sc-374022; RRID: AB_10916877
JC-D7	MedKoo	Cat# 585048
JC-D8	MedKoo	Cat# 463766
Lamin A/C	Santa Cruz Biotechnology	Cat# sc-376248; RRID: AB_10991536
Myc	Santa Cruz Biotechnology	Cat# sc-40; RRID: AB_627268
NOP2	Invitrogen	Cat# PA5-59073; RRID: AB_2644641
Nucleolin	Santa Cruz Biotechnology	Cat# sc-17826; RRID: AB_670270
Nucleophosmin	Abcam	Cat# ab10530; RRID: AB_297271
PAF49	Invitrogen	Cat# MA5-27813; RRID: AB_2735299
Pericentrin	Abcam	Cat# ab4448; RRID: AB_304461
Secondary horseradish peroxidase-conjugated	Santa Cruz Biotechnology	Cat# sc-516102; RRID: AB_2687626
Secondary horseradish peroxidase-conjugated	Sigma	Cat# GENA934; RRID: AB_2722659
SYTO™ RNASelect™ Green Fluorescent cell Stain	Thermo Fisher Scientific	Cat# S32703

**Bacterial and virus strains**		

*E. coli* DH10B	Invitrogen	Cat# 18297010

**Chemicals, peptides, and recombinant proteins**		

1,6-Hexanediol	Sigma	Cat# 240117
2% aqueous Osmium tetroxide solution	TAAB	Cat# O005
500× Protease inhibitor cocktail	Sigma	Cat# P8340
AccuGel 19:1, (40% Acrylamide: Bis-Acrylamide 19:1)	Geneflow	Cat# A2-0060
Albumin	Sigma	Cat# A2153
BrUTP	Sigma	Cat# B7166
Doxycycline Hydrochloride	Sigma	Cat# D3072
Dulbecco’s Modified Eagle Medium (DMEM)	Gibco	Cat# 21969035
Epon 812 resin Kit	TAAB	Cat# T043
Fetal Bovine Serum (FBS)	Gibco	Cat# 10500064
Fibronectin	Sigma	Cat# F1056
Formaldehyde 36% stock EM grade	TAAB	Cat# F003
GlutaMAX™	Gibco	Cat# 35050087
Glut 25% stock vials	TAAB	Cat# G011
His-PPPX1	Lonetti et al. 2011^48^	N/A
IMM mounting medium	Ibidi	Cat# 50001
NuPAGE 4–12% bis-tris gels	Invitrogen	Cat# NP0321
P100	RegeneTiss Co.	N/Aa
poly-L-ornithine hydrobromide	Sigma	Cat# P3655
ProLong™ Diamond Antifade Mountant	Invitrogen	Cat# P36970
Propylene glycol	Sigma	Cat# W294004
PVDF membrane	Bio-Rad	Cat# 1620177
Recombinant polyP binding domain (PPBD)	Saito et al., 2005^47^	N/A
S-(5′-Adenosyl)-L-methionine iodide	Sigma	Cat# A4377
Sulforhodamine B	Sigma	Cat# S1402
Titanium dioxide beads	Hichrom	Cat# 5020-75000
Triton X-100	Sigma	Cat# T8532

**Critical commercial assays**		

Clarity Max™	Bio-Rad	Cat# 1705062
DC Protein Assay	Bio-Rad	Cat# 5000116
Lipofectamine 2000	Thermo Fisher Scientific	Cat# 11668027
Lipofectamine 3000	Thermo Fisher Scientific	Cat# L3000001

**Deposited data**		

Raw data	This paper	https://doi.org/10.17632/cwmfs4jwtk.1

**Experimental models: Cell lines**		

HeLa	ATCC	Cat# CRM-CCL-2; RRID: CVCL_0030
HTC116	ATCC	Cat# CCL-247; RRID: CVCL_0291
Human embryonic kidney (HEK) 293	ATCC	Cat# CRL-1573; RRID: CVCL_0045
Human retinal pigment epithelial-1 (RPE1)	ATCC	Cat# CRL-4000; RRID: CVCL_4388
N2A	ATCC	Cat# CCL-131; RRID: CVCL_0470
T-REx™-293 Cell Line	Invitrogen	Cat# R71007; RRID: CVCL_D585

**Recombinant DNA and primers**		

pCMV-Myc-N	Clontech	Cat# 635689
pGEM®-T Easy Vector Systems	Promega	Cat# A1360
Primer OC440: ccg ctcgag a ATGGGTCA GGAAAAGCTATACATCG∗	N/a	5′ XhoI-EcPPK (AP_003087)
Primer OC441: ataagaat GCGGCCGC TTATT CAGGTTGTTCGAGTGATTTGATG∗	N/a	3′ NotI-EcPPK (AP_003087)
pTRE3G	Clontech	Cat# 631173

**Software and algorithms**		

Amira	Thermo Scientific	Version 2019
AZtec	Oxford Instruments	Version 2.1
Epson scan	Epson	Version 3.04A
Fiji	ImageJ	N/A
Photoshop	Adobe	Version CC 2019
Prism	GraphPad Software	Version 9

### Resource availability

#### Lead contact

Further information and requests for resources and reagents should be directed to the lead contact, Adolfo Saiardi (a.saiardi@ucl.ac.uk).

#### Materials availability

The TREx-PPK1 cell line and all reagents generated in this study are available from the lead contact.

#### Data and code availability

• Any raw files or information required to reanalyse the data reported in this paper is available to download from Mendeley Data (https://doi.org/10.17632/cwmfs4jwtk.1) or from the lead contact upon request.
• This paper does not report any original code.
• Any additional information required to reanalyse the data reported in this paper is available from the lead contact upon request.

### Experimental model and subject details

#### Establishment of inducible TREx-PPK1 cells

HEK293 T-REx (Invitrogen, Life Technologies) were seeded into wells in a 6-well plate at 5x10^4^, 1x10^5^, 5x10^5^ and 1x10^6^ cells/mL. The well that reached 70% confluency, was co-transfected with 1 μg of ScaI linearized pTRE3G (Clontech) and 150 ng of puromycin vector with Lipofectamine 2000 Transfection Reagent (5:1) according to the manufacturer’s instructions (Thermo Fisher Scientific, #11668027). Non-transfected cells were used throughout the procedure as control. Next day, cells were trypsinized and 10 mL DMEM (Gibco, #21969035) media was added. Cells were counted (7.1 x 10^5^ cells/mL) and diluted into four 15 cm^2^ plates as follows: Plate (1) 50 μL of cells plus 20 mL of DMEM, Plate (2) 200 μL of cells plus 18.8 mL of DMEM, Plate (3) 2 mL of cells plus 18 mL of DMEM and Plate (4) 5 mL of cells plus 15 mL of DMEM. 6 μL of 1 μg/ml Puromycin was added to Plates (1) and (2), 6.6 μL to Plate (3) and 7.5 μL to Plate (4). Cells were incubated for a week, after which they were washed twice with PBS, and 20mL of fresh DMEM media with 6 μL of Puromycin (1 μg/ml) was added. No cells were recovered from the non-transfection control. Over a couple of weeks, approximately 80 individually growing colonies were trypsinized and seeded into 24-well plates, where they were further selected in DMEM with puromycin as before. Once confluent, cells were split in three: one for stock and two for screening. The screening was performed in 12-well plates (total volume of 2 mL per well). To induce *Ec*PPK1 expression, 1 μL of 2 mg/mL doxycycline was added to one of the two wells of each colony and the other well was kept uninduced as control. Twenty-four hours post-Doxy induction, cells were trypsinized, washed with PBS, scraped, pooled in threes, and proteins were extracted with RIPA buffer. Protein extracts were run on NuPAGE and western blotted with anti-Myc antibody as described below. Pools of induced cells showing the 89 kDa band corresponding to Myc-*Ec*PPK1 were kept. A total of 2 positive clones were recovered. It is important to note that this protocol was first attempted with plasmid pCA429 in which *Ec*PPK1 is under the expression of the strong CMV constitutive promoter. However, despite screening almost 100 colonies, no transgenic cells were recovered most likely due to lethality, hence the change of strategy by using an inducible promoter. Even under inducible conditions only 2 cell lines were recovered out of almost 80 screened.

### Method details

#### Cloning

*Escherichia coli* PPK1 (based on K-12 substr. W3110, locus AP_003087) was amplified from *E. coli* DH10B genomic DNA with the primers OC440/OC441 (STAR Methods table) and cloned into pGemT-easy (Promega), originating from construct pCA319. Construct pCA319 was digested with XhoI/SalI and ligated into pCMV-Myc-N (Clontech) digested with the compatible enzymes SalI/NotI, originating construct pCA429. Finally, construct pCA429 was digested with the enzymes PspOMI/EglI and ligated into pTRE3G digested with the same enzymes, originating construct pCA550. The pTRE3G Vector provides a tightly regulated inducible mammalian expression system responsive to Tet-On, Tet-Off, and Tet-Express transactivators.

#### Cells culture and transfections

TREx-PPK1 #1, Human embryonic kidney (HEK) 293, HeLa, HTC116, N2A and Human retinal pigment epithelial-1 (RPE1) cells were grown and maintained in DMEM (Gibco, #21969035) supplemented with 10% fetal bovine serum (Gibco, #10500064) and 1% GlutaMAX (Gibco, #35050087), in a humidified cell culture incubator, under a 5% CO_2_ atmosphere, at 37°C. For image analysis, cells were plated on glass coverslips in a 4-well plate mounted with fibronectin (Sigma, #F1056) diluted at 1:100 in Phosphate-buffered saline (PBS) for 1 h at 37°C. All treatments and transfections started after 24 h of incubation. TREx-PPK1 cells were induced with 1 μg/ml doxycycline. HEK and HeLa cell cultures (70%–80% confluent) were transfected with the pCMV-MycPPK1 plasmid using the Lipofectamine 3000 transfection reagent (Thermo Fisher Scientific, #L3000001), following manufacturer’s instructions. Transfection was performed for 5 h at 37°C in a 5% CO_2_ incubator and used after 48 h of transfection.

#### Cell growth assay

The cell growth was analyzed using sulforhodamine B (SRB).^81^^,^^82^ Cells were seeded into 96 well plates. After 24 h, the cells were washed and 100 μL of media containing 1 μg/mL doxycycline was added. At every timepoint, cells were fixed in 10% trichloroacetic acid and stained with 0.05% sulforhodamine B (Sigma, #S1402) solubilized in 10mM Tris base in 1% acetic acid. The absorbance was measured on a Tecan plate-reader fluorimeter at 500nm. The results are expressed as growth percentage (%) relative to the untreated control cells.

#### Light microscopy for toluidine blue

Cells were washed twice with PBS and fixed in 4% cold paraformaldehyde solution for 10 min at room temperature (RT). Afterward, cells were washed twice with Milli-Q water and incubated with 0.05% toluidine blue for 10 min. Cells were then washed thrice with Milli-Q water and mounted using IMM mounting medium (Ibidi, #50001). Bright, phase contrast, and dark field images were observed under Olympus CX41 light microscope using a 40× objective magnification. Images were captured using a GXCAM U3 18MP camera attached to an Olympus Microscope via a U-CMAD3 C Mount Adapter and a U-TV1X-2 camera adapter, utilizing GT Vision GXCapture-T software. Dark field microscopy is background-free scattering-based microscopy that directly detects scattering from a sample by rejecting excitation light.^83^ For PPX1 experiments, TREx-PPK1 #1 cells were incubated with recombinant His-PPPX1 in reaction buffer (200mM HEPES pH 6.8; 60mM MgSO4; 1M KCl; 10mM DTT) for 1 h at 37°C. Permeabilised cells were pre-treated with 0.1% Triton X-100 in PBS for 15 min.

#### Confocal microscopy

Cells were washed twice with PBS and fixed in 4% cold paraformaldehyde solution, in cold methanol for SYTO RNASelect Green stain, or in pericentrin immunohistochemistry for 10 min at RT. Cells were then washed twice with PBS followed by permeabilization solution (0.1% Triton X-100 in PBS) for 10 min at RT. Next, blocking solution (3% BSA in PBS) was added to the cells and kept for 1 h at RT. Cells were later probed with primary antibodies for myc (Santa Cruz Biotechnology, #sc-40), lamin A/C (Santa Cruz Biotechnology, #sc-376248), fibrillarin (Santa Cruz Biotechnology, #sc-374022), nucleophosmin (Abcam, #ab10530), nucleolin (Santa Cruz Biotechnology, #sc-17826), PAF49 (Invitrogen, #MA5-27813), SYTO RNASelect Green Fluorescent cell Stain (Thermo Fisher, #S32703), NOP2 (Invitrogen, #PA5-59073), pericentrin (Abcam, #ab4448), JC-D7 (MedKoo, #585048), JC-D8 (MedKoo, #463766), DDX6 (Invitrogen, #PA5-27786), diluted at 1:200 in blocking buffer for 1 h at RT. Next, cells were washed twice for 5 min with PBS and labeled with Alexa Fluor 488 (Invitrogen) at 1:2000 dilution in blocking buffer for 1 h at RT. Next, cells were stained with 300nM 4′,6-Diamidino-2-Phenylindole (DAPI) (Sigma, #D9542) and mounted on glass slides with ProLong Diamond Antifade Mountant (Invitrogen, #P36970) for fluorescence microscope imaging. DAPI polyP images were observed under an Opera Phoenix high-content confocal microscope using a 63× water immersion. Other confocal analyses were performed using the Olympus Fluoview FV1200 confocal microscope and a 60× oil immersion lens.

#### polyP binding assay for PPBD

Recombinant polyP binding domain (PPBD) with *E. coli* PPX linked with an Xpress epitope tag was prepared as described by Saito et al.^47^ Cells were washed twice with PBS and fixed in 4% cold paraformaldehyde solution for 10 min at RT. Then, cells were washed with PBS for 5 min followed by two washes of TBS (pH 8.3) for 5 min each. Next, the cells were incubated with permeabilization solution (0.1% Triton X-100 in TBS pH 8.3) for 2 min at RT. After, the cells were washed twice with TBS pH 8.3 and then the blocking solution (3% BSA in TBS pH 8.3) was added for 1 h at RT. The solution was replaced with a new blocking solution with 20 μg/ml of PPBD for 1 h. After this time, the cells were washed twice with TBS pH 8.3 for 5 min and incubated with 2 μg/ml of Anti-Xpress (Invitrogen, #R910-25) in blocking solution for another 1 h. Next, the cells were washed with 0.05% Triton X-100 in TBS pH 8.3 and twice with TBS pH 8.3. Next, the cells were labeled with Alexa Fluor 633 (Invitrogen) at 1:2000 dilution in blocking solution for 1 h at RT. Then, the cells were, in turn, washed once with 0.01% Triton X-100 in TBS pH 8.3, twice with TBS pH 8.3 and once with PBS. Next, the cells were stained with 300nM DAPI and mounted on glass slides with ProLong Diamond Antifade Mountant for fluorescence microscope imaging. The PPBD images were observed under an Olympus Fluoview FV1200 confocal microscope using a 60× oil immersion lens.

#### PolyP extraction using phenol extraction

PolyP extraction using phenol extraction was performed in accordance with the protocol by Bru et al. with some modifications.^84^ For this extraction, cells were seeded into one 90mm dish. When ready, the cells were washed twice with cold PBS and then scrapped with 250 μL of PBS. The cells were then centrifuged at 2,400 × g for 1 min and the supernatant was discarded. The pellet was resuspended in 250 μL of LETS buffer (100mM LiCl, 10mM EDTA, 10mM Tris-HCl pH 7.4, 0.2% SDS) and 250 μL of phenol pH 4.8, then vortexed for 1 min and placed on ice for at least 1 min. The mixture was then centrifuged at 1,600 × g for 5 min and the supernatant was transferred to a new tube containing 250 μL chloroform. This was vortexed for 1 min and centrifuged at 9,500 × g for 5 min. The top layer was transferred to a new tube with 300 μL of absolute ethanol cooled to −20°C was added to the tube which was then incubated in dry ice for 30 min. The tubes were then centrifuged at 17,100 × g for 15 min, the supernatant was discarded and the tube was left open to dry the pellet at RT until completely dry. Finally, the pellet was resuspended in 100 μL of Milli-Q water for further assays.

#### PolyP extraction using titanium dioxide beads

PolyP extraction using titanium beads was performed in accordance with the protocol proposed by Wilson and Saiardi et al. with some modifications.^85^ For this extraction, cells were seeded into one 90mm dish. When ready, the cells were washed twice with cold PBS and 500 μL of 1M perchloric acid was added. The dish was then incubated for 10 min with 1-min rotations. The liquid was collected and centrifuged at 2,400 × g for 1 min to remove the debris. The samples were transferred to new tubes and 4 mg of TiO_2_ nanobeads (Hichrom, #5020–75000) were added. After 20 min of rotation at 4°C, the tubes were centrifuged at 2,400 × g for 1 min at 4°C. The supernatant was carefully discarded and then the pellet was washed twice by resuspending in 500 μL 0.1M perchloric acid and then centrifuged at 2,400 × g for 1 min at 4°C. After this step, the pellet was resuspended twice in 200 μL–2.8% ammonium hydroxide, vortexed and then rotated for 5 min at 4°C. After each elution with ∼2.8% ammonium hydroxide, the supernatant was transferred to a new tube and to finish a centrifugal evaporator was used to reduce the sample volume to 20-40 μL.

#### polyP analysis using PAGE

Polyacrylamide gel electrophoresis (PAGE) was performed as described by Losito et al. with some modifications.^86^ The samples were resolved using a 30% polyacrylamide gel in TBE (28.5 mL 40% Acr/Bis (19∶1); 3.8 mL 10× TBE; 3.8 mL H_2_0; 200 μL 10% APS; 15 μL TEMED) sized 24 × 16 × 0.1cm. The gels were pre-run for 30 min at 660 V. 10 μL of 4× Dye (10mM TrisHCl pH 7.0; 1mM EDTA; 30% glycerol; 0.1% Orange G) was added to each sample prior to loading onto the gels. Gels were run at 660 V overnight at 4°C until the Orange G (OG) dye front reached 10 cm from the gel’s bottom. For DAPI staining, the gels were gently agitated for 30 min at RT in staining solution (20% methanol; 1% glycerol; 20mM Tris base; 200 μL DAPI 10 mg/mL) and then destained for 30 min in the same solution without DAPI. The gels were exposed to 300 nm light using a NuGenius UV transilluminator (Syngene) for 1–4 min to induce photobleaching, after which photographs were taken. For toluidine staining, gels were gently agitated for 30 min at RT in a filtered staining solution (20% methanol; 2% glycerol; 0.05% Toluidine Blue). To distain, the gels were gently agitated for 90 min at RT in the same solution without the Toluidine Blue, refreshing this whenever visibly saturated. Pictures were taken after exposing the gel on a white light transilluminator. For the toluidine metachromatic gel, we used the ‘color restoration’ feature from the Epson scan software.

#### Nucleoli isolation

The nucleolar isolation method was performed in accordance with the protocol proposed by Li and Lam et al.^52^ Cells were washed twice with precooled solution S-I (0.5M sucrose, 3mM MgCl_2_) at −20°C, scraped and transferred to a new tube. The cells were sonicated on ice at 25% power, 10 s on, 10 s off, for five cycles. The sonicated cell suspension was transferred to a tube containing 0.7 mL of precooled S-II (1M sucrose, 3mM MgCl_2_) and centrifuged at 1,800 × g for 5 min at 4°C in a swinging-bucket centrifuge. The resulting pellet containing the isolated nucleoli was resuspended in 100 μL of Milli-Q water and the supernatant was transferred to a new tube for further assays.

#### Isolated nucleoli microscopy

To attach the isolated nucleoli into coverslips, we prepared 4-well plates with coverslips and incubated them with 100% ethanol for 5 min at RT. We then twice washed the coverslips with Milli-Q water and incubated them with 0.1 mg/mL poly-L-ornithine hydrobromide (Sigma, #P3655) solution in water for 10 min at RT. After the nucleoli isolation, one aliquot was diluted in water (1:2) and added directly to the coated coverslips. The plate was centrifuged for 5 min at 450 × g, 4°C. The coverslip was washed with Milli-Q water and then fixation and toluidine blue staining followed as earlier described.

#### Protein extraction

Cells were washed twice with PBS, scraped and centrifuged for 1 min at 2,400 × g to remove the debris. The PBS was then removed and we added 100 μL of lysis buffer (50mM Tris-HCl pH 8, 150mM NaCl, 5mM DTT, 0.5% Triton X-100) with a 100× Phosphatase inhibitor (200mM imidazole, 100mM sodium fluoride, 115mM solidum molybdate, 100mM sodium orthovanadate) and 500× Protease inhibitor cocktail for mammalian cells (Sigma, #P8340). The samples were resuspended and rotated for 5 min at 4°C and then centrifuged for 5 min at 17,100 × g. The supernatant was collected in a new tube for further assays.

#### Western blotting

Protein concentrations in protein extracts and nucleoli isolation were measured using the DC Protein Assay (Bio-Rad, #5000112). Equal amounts of protein extraction (20-50 μg) were resolved using NuPAGE 4–12% bis-tris gels (Invitrogen, #NP0321) and proteins were transferred to PVDF membranes (Bio-Rad, #1620177). Membranes were blocked for 1 h using 5% non-fat milk blocking solution (100nM NaCl, 10mM Tris HCl pH 7.5, 0.1% Tween 20) then blotted overnight for the following primary antibodies at 1:1000–1:2000 in 1% albumin (Sigma, #A2153): lamin A/C (Santa Cruz Biotechnology, #sc-376248), fibrillarin (Santa Cruz Biotechnology, #sc-374022), nucleophosmin (Abcam, #ab10530), nucleolin (Santa Cruz Biotechnology, #sc-17826), PAF49 (Invitrogen, #MA5-27813), NOP2 (Invitrogen, #PA5-59073) and α-tubulin (Biolegend, #625902). Secondary horseradish peroxidase-conjugated antibodies (Santa Cruz Biotechnology, #sc-516102; Sigma, #GENA934) were diluted in 3% albumin. Immunocomplexes were detected using a luminol peroxidase chemiluminescence kit (Clarity Max, Bio-Rad, #1705062) and acquired using the Alliance Q9, UVITEC imaging system. Protein band intensity was quantified using ImageJ software (National Institutes of Health, Bethesda, MD, USA).

#### PolyP quantification using the malachite green assay

PolyP was measured as free phosphates following enzymatic digestion of the polymer. To degrade polyP to free phosphate groups, the different extracts were incubated with recombinant exopolyphosphatase His-PPX1^48^ in reaction buffer (200mM HEPES pH 6.8; 60mM MgSO_4_; 1M KCl; 10mM DTT) for 1 h at 37°C. Samples (2–10 mL) were dispensed in triplicate on a 96-well plate and the volume adjusted to 100 μL with H_2_O. Then 100 μL of freshly mixed Molybdate (175mM (NH_4_)2MoO_4_; 2M H_2_SO_4_) and Malachite (0.127 malachite green; 1.4g polyvinyl alcohol (100,000 MW) in 400mL H_2_O) solution prepared as a 4:3 ratio was added to each well. The absorbance was measured on a Tecan plate-reader fluorimeter at 600 nm and compared with a sodium phosphate standard curve. All results were normalized using RNA or protein quantification. The results presented come from three or more independent experiments.

#### Run-on transcription assay

The run-on assay was performed in accordance with the protocol proposed by Wansink et al.^87^ with some modifications. Briefly, the cells were cultured on coverslips as described previously. When ready, the coverslips were washed once with TBS and twice with glycerol buffer (20mM Tris-HCl pH 7.4, 5mM MgCl_2_, 25% glycerol, 0.5mM PMSF, 0.5mM EGTA). Then the cells were permeabilised with glycerol buffer containing 0.05% Triton X-100 for 3 min at RT. The glycerol buffer was then removed and a transcription buffer (100mM KCl, 50mM Tris-HCl pH 7.4, 5mM MgCl_2_, 25μM S-(5′-Adenosyl)-L-methionine iodide (Sigma, #A4377), 5U/ml RNase inhibitor, 1mM PMSF) containing 0.5mM of ATP, CTP, GTP and 0.2mM BrUTP (Sigma, #B7166) was added and incubated for 30 min at RT. The coverslips were then washed once with TBS containing 0.5% Triton X-100 and 5 units/ml RNase inhibitor for 3 min, and once with TBS containing 5 U/ml RNase inhibitor. Cells were fixed immediately afterward as earlier described.

#### Quantitative image analysis

The net fluorescence intensity for the SYTO RNASelect Green Fluorescent Cell Stain, the Run-On transcription assay, and the quantification of PAF49 spots per nucleus were acquired using ImageJ. In brief, each cell was outlined using the freehand ROI (regions of interest) tool for fluorescence intensity quantification. After delineating each cell, the mean intensity was obtained using the ImageJ software. The mean intensity of the background area was then subtracted from all mean intensity values to produce final results. For the quantification of PAF49 spots, we counted the number of spots per nucleus. Each of the two channel images was opened separately, and a threshold was applied. Then, under the ‘set measures’ tool, the following options were selected: ‘area’, ‘bounding rectangle’, ‘area fraction’, and ‘limit to threshold’. The DAPI channel image was analyzed first using the ‘analyze particles’ tool with the minimum size set to 10 μm^2^ to avoid measuring objects smaller than the nucleoli. This created ROI shapes, on this occasion nuclei, which can be managed through the ROI manager. With the ROI manager opened, the PAF49 channel image was opened and the ‘analyze particles’ tool was used again this time to analyze the number of puncta inside each nucleus with these then exhibited in the summary.

#### Nucleolar size analysis

DAPI immunofluorescence images were used to calculate the nucleolar area, and the signal was quantified through the utilisation of Fiji (ImageJ) software. Briefly, nuclear regions of interest (ROIs) were defined in the DAPI channel, using Thresholder Blur, Make Binary, Fill Holes and Watershed followed by hand segmentation to ensure accurate ROIs. Image background signal was determined by defining five non-cellular ROIs per image and averaging the pixel intensity. This background was subtracted from all ROI data.

#### rRNA analysis

RNA was extracted from control or induced cells, or from nucleolar pellet purified from 80% confluent 90mm dish. PBS washed cells pellet or nucleolar pellet was resuspended in 250 μL of LETS buffer (100mM LiCl, 10mM EDTA, 10mM Tris-HCl pH 7.4, 0.2% SDS). Then 250 μL of phenol pH 4.8 was added and the samples were vortexed for 1 min and placed on ice for at least 1 min. After centrifugation of the mixture at 16,000 × g for 5 min, the supernatant was transferred to a new tube containing 250 μL chloroform, vortexed for 1 min and centrifuged at 9,400 × g for 5 min. The RNA in the recovered supernatant was precipitated with 2 volumes of 100% ethanol incubating at −20°C overnight. After spinning for 10 min at 16,000 × g the RNA pellet was air dry, resuspended in 100 mL Milli-Q water and quantified using a nanodrop Spectrophotometer (Thermo Fisher). A denaturing agarose gel electrophoresis was utilised to resolve rRNAs. The 1% agarose gel was prepared with 4% formaldehyde in 1× MOPS buffer (20mM MOPS pH7.0, 5mM Sodium acetate, 1mM EDTA). RNA (20 mg) was resuspended in denaturing loading buffer (50% Formamide; 4% Formaldehyde; 25% Glycerol; 1× MOPS buffer; 1 mg/mL Ethidium Bromide; trace of Bromophenol blue dye) and heated for 5 min at 75°C before loading into the gel. The gels were run at 100 V at 4°C, in running buffer (1× MOPs buffer; 2% Formaldehyde), until the bromophenol blue front run for 13–15 cm. The gel images were acquired using a NuGenius UV transilluminator (Syngene).

#### Transmission electron microscopy (TEM)

Cell coverslips or isolated nucleoli, were fixed with EM grade 2% formaldehyde, and 1.5% glutaraldehyde in 0.1M sodium cacodylate at pH 7.4 for 30 min before secondary fixation with 1% osmium tetroxide and 1.5% potassium ferricyanide at 4°C for 1 h. After a prolonged wash in 0.1M sodium cacodylate, samples were treated with 1% tannic acid in 0.05M sodium cacodylate for 45 min in the dark at RT, then subsequently dehydrated with two incubations of 5 min in 70%, then 90%, then 100% ethanol. Samples were then incubated with a 50:50 mixture of Propylene Oxide: Epon 812 resin (TAAB), for 1 h at RT, before two further exchanges with 100% Epon 812 resin for 1.5–2 h each in a fume hood. Ultrathin sections (70nm thick) were collected on formvar coated slot grids. Grids were either imaged unstained or stained with Waltons Lead citrate prior to imaging in the TEM (120kV Tecnai Spirit BioTwin, FEI, Thermofisher), see figure legends for details.

#### Volume EM using array tomography scanning electron microscopy (AT-SEM)

Cells were fixed and treated with reduced osmium as for TEM, before being incubated sequentially with 1% thiocarbohydrazide (15 min at RT), 2% osmium tetroxide (15 min at 4°C), 1% uranyl acetate (15 min at 4°C), and 0.7% of lead aspartate (15 min at 60°C) with Milli-Q water washes in between. Samples were then dehydrated through an ethanol series and embedded in Epon 812 resin. Serial ultrathin sections (70nm thick) were generated using an ultramicrotome (Leica UC7) and 45° Ultra Jumbo Diamond knife (Diatome) and collected on ITO coated coverslips (Diamond coatings UK) using the paper clip method.^88^ Serial sections were imaged in a FEG-SEM (Zeiss Gemini 300), with an acceleration voltage of 4.5kV, landing energy 1.5kV, using the Sense-BSD detector (Zeiss) at 2nm or 5nm pixel size and a working distance of 5mm.

#### 3D reconstruction and quantification of volume EM data

Images of serial sections were down sampled to 10nm pixels, aligned using TrakEM2^89^ in Fiji^90^ and imported into Amira^91^ for manual segmentation. All surfaces were generated using unconstrained smoothing (factor 5) and surface volume were quantified using Surface Area Volume module for materials.

#### Energy Dispersive X-Ray spectroscopy

Cells were fixed with 2% formaldehyde, 1.5% glutaraldehyde in 0.1M PIPES pH 7.2 for 30 min at RT. Samples were then washed with 0.1M PIPES pH7.2 before being incubated at 4°C overnight with 0.5% Uranyl Acetate prepared in distilled water. Samples were then dehydrated and embedded in Epon as described above for TEM. Sections (70nm thick) were then collected onto formvar coated slot grids and analyzed unstained. Grids were loaded into the STEM grid holder of a Zeiss Sigma 300 FEG-SEM and imaged at an accelerating voltage of 8kV with a 60 μm aperture and a working distance of 5.4mm, using the back scattered electron detector in high gain mode. Elemental analysis was performed using an Ultim Extreme detector (Oxford Instruments) and Aztec analysis software with a 2048 x 2048 frame size, a process time of 5 and a pixel dwell of 10 μs. The spectra were collected from at least 3 nucleoplasm and 3 nuclear inclusion (e.g.,: nucleoli and electron dense structures) sub-domains using the Point & ID function for each cell, with 9 replicate cells for each non-induced and induced conditions. Extraneous elements (Al, Cu, Si, S, Na and K) were removed from the analysis. The percentage weight of each element per sub-domain was averaged for each cell and this value was used in subsequent statistical analyses of the total pool of 9 cells per cell line.

#### PolyP coacervates analysis

Calcium chloride (CaCl_2_, 10mM) and magnesium chloride (MgCl_2_, 10mM) solutions were prepared and subsequently incubated with a 20mM Tris-HCl buffer at RT. To these solutions, a predefined quantity of polyphosphate (polyP_100_) was added. The mixture was incubated for 5 min to ensure adequate interaction between the components. Following the incubation period, the turbidity of each solution was measured at a wavelength of 350 nm. This assessment aimed to determine the extent of complex formation in the solution. PolyP coacervates, produced in experiments buffered at pH 8.5, were carefully transferred (20 μL) onto microscopy slides. A coverslip was gently placed over the sample, and the slides were examined using both dark field (DF) and phase contrast (PH) microscopy techniques.

#### Liquid-liquid phase separation assay

TREx-PPK1 cells were induced for 48 h. After the induction, cells were treated with 4% (v/v) propylene glycol (PG) (Sigma, #W294004) or 4.7% (v/v) 1,6-hexanediol (1,6HD) (Sigma, #240117) in DMEM medium. Cells were then returned into the CO_2_ incubator at 37°C for additional 60 min incubation. After incubation, the cells were fixed immediately and dyed with toluidine as previously described for image analysis.

### Quantification and statistical analysis

#### Statistical analysis

Statistical results are expressed as the mean ± SEM, *n* ≥ 3. The normality was confirmed by Shapiro-Wilk test. Unpaired Student’s t-test for parametric and Mann-Whitney for nonparametric data was performed using Graph Pad Prism 9 (Graph Pad Software, San Diego, USA). The acceptance level of significance was set at *p* < 0.05. The experiments statistical details can be found in the figure legends.

## Supplemental information

Document S1. Figures S1‒S5

Document S2. Article plus supplemental information
