# Supplementary material for: A mammalian model reveals inorganic polyphosphate channeling into the nucleolus and induction of a hyper-condensate state
Source: Cell Rep Methods. 2024 Jul 8;4(7):100814. doi: 10.1016/j.crmeth.2024.100814 (PMC11294840; doi:10.1016/j.crmeth.2024.100814)
Supplement: Document S1. Figures S1‒S5 [file mmc1.pdf]

Cell Reports Methods, Volume *4*

Supplemental information

A mammalian model reveals inorganic polyphosphate channeling into the nucleolus and induction of a hyper-condensate state

Filipy Borghi, Cristina Azevedo, Errin Johnson, Jemima J. Burden, and Adolfo Saiardi

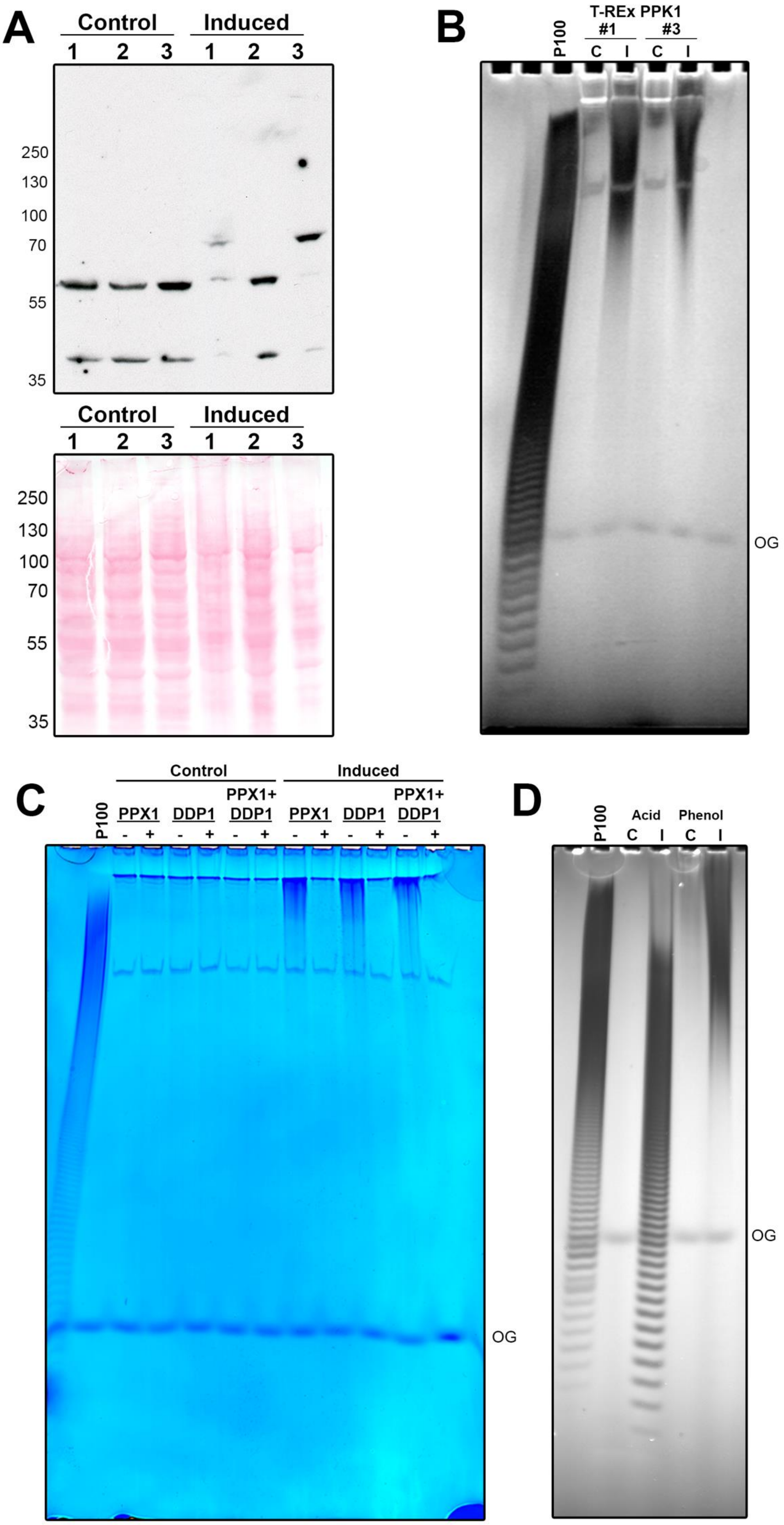

A
Control
Induced
1 2 3 1 2 3
250
130
100
70
55
35
B
P100
T-REx PPK1
#1
#3
C I C I
OG
C
Control
Induced
P100
PPX1
DDP1
PPX1+ DDP1
- +
OG
D
P100
Acid
Phenol
C I C I
OG

Supplemental Figure 1 – Establishment of Inducible TREx-PPK1 lines. Related to Figure 1.

Anti-Myc Western blot analysis for clones #1 to#3, before and after induction with 1µg/mL doxycycline (**A**). Induced clones #1 and #3 express a band migrating above the 70 kDa marker, which is in line with the expected size of the tagged Myc-EcPPK1 (top). Ponceau S staining demonstrated similar loading (bottom). PAGE analysis (**B**) of phenol-extracted polyP/RNA from control (C) and doxycycline-induced (24 hours) (I) T-REx PPK1 clones #1 and #3. RNA (10µg) from extracts was resolved on 30% PAGE and stained with DAPI. (**C**) PAGE gel of phenol-extracted polyP from control (C) and doxycycline-induced (24 hours) (I) TREx-PPK1 #1. PolyP/RNA (10µg) extracts were treated with exopolyphosphatase (PPX1), diphosphoinositol polyphosphate phosphohydrolase (DDP1), or a combination of both (PPX1 + DDP1), resolved on 30% PAGE, and stained with Toluidine Blue. Orange G (OG) was used as a migration standard, while polyP$_{100}$ (P100) was used to orient the gel. DAPI photobleaching did not reveal endogenous polyP (**D**). PAGE analysis of acid/TiO_2 (10% of extract) and phenol-extracted (10µg of RNA) polyP from control (C) and cells induced for 48 hours (I).

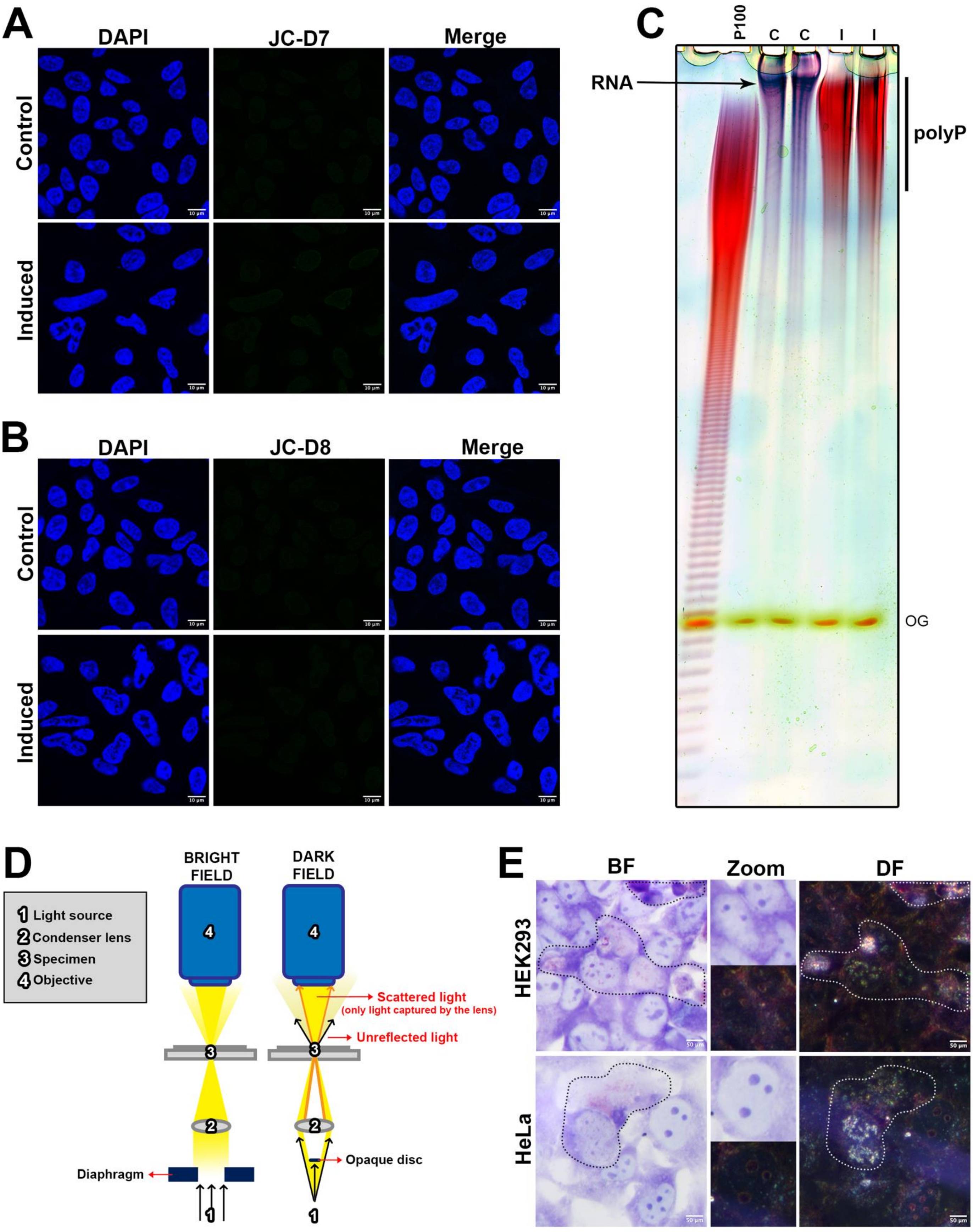

A
DAPI
JC-D7
Merge
Control
Induced
B
DAPI
JC-D8
Merge
Control
Induced
C
P100
C
C
I
I
RNA
polyP
OG
D
BRIGHT FIELD
DARK FIELD
1 Light source
2 Condenser lens
3 Specimen
4 Objective
Scattered light
(only light captured by the lens)
Unreflected light
Diaphragm
Opaque disc
E
BF
Zoom
DF
HEK293
HeLa

Supplemental Figure 2 – polyP detection methods by microscopy. Related to Figure 2.

Confocal immunofluorescence analysis was conducted to detect polyP using JC-D7 (**A**) and JC-D8 (**B**), both in green, with the nucleus stained with DAPI (blue). Toluidine-polyP metachromasia (**C**). PAGE analysis of phenol-extracted polyP/RNA from induced TREx-PPK1 cells. After staining with toluidine blue, true colours were revealed by subtracting the background using the colour restoration feature of the Epson scanning software. The metachromasia of toluidine allows it to change colour depending on the type of ligand. We observed that polyP exhibited a pinkish/reddish colour, while RNA stained blue. Comparison of light paths in transmission light microscopy and dark-field microscopy (**D**). Bright-field microscopy, illustrated on the left, allows light to pass directly through the specimen, producing a bright background. Dark-field microscopy, illustrated on the right, employs a light-blocking disk just below the condenser. This blockage allows only edge light to pass through. The edge light enters the objective only if it is scattered by encountering a differentially dense element. Bright (BF) and dark-field (DF) images of transfected HEK293 and HeLa cells after toluidine blue staining are shown in (**E**). The cells were transiently transfected with pCMV-MycPPK1 and observed 48 hours post-transfection.

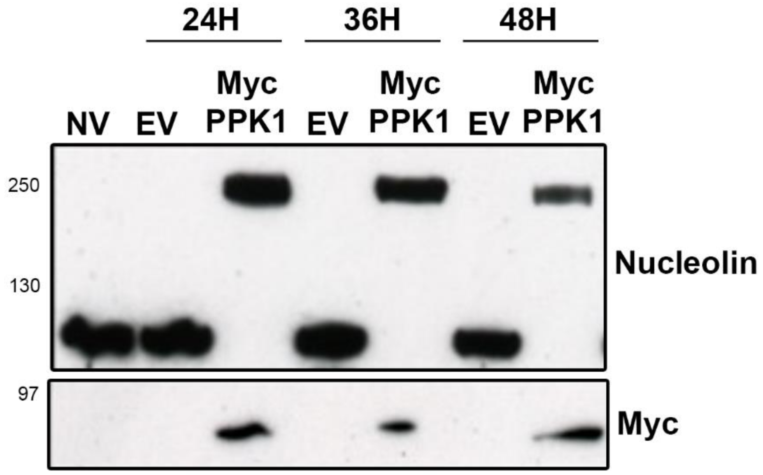

Supplemental Figure 3 – Nucleolin mobility shift in *Ec*PPK1 transient transfected cells. Related to Figure 3.

The HEK293 cells were transiently transfected with pCMV-MycPPK1, and protein extracts were prepared at the indicated times. Western blot analysis with anti-nucleolin revealed a dramatic mobility shift in the cells expressing EcPPK1, as detected using an anti-Myc antibody. NV = no vector; EV = empty vector.

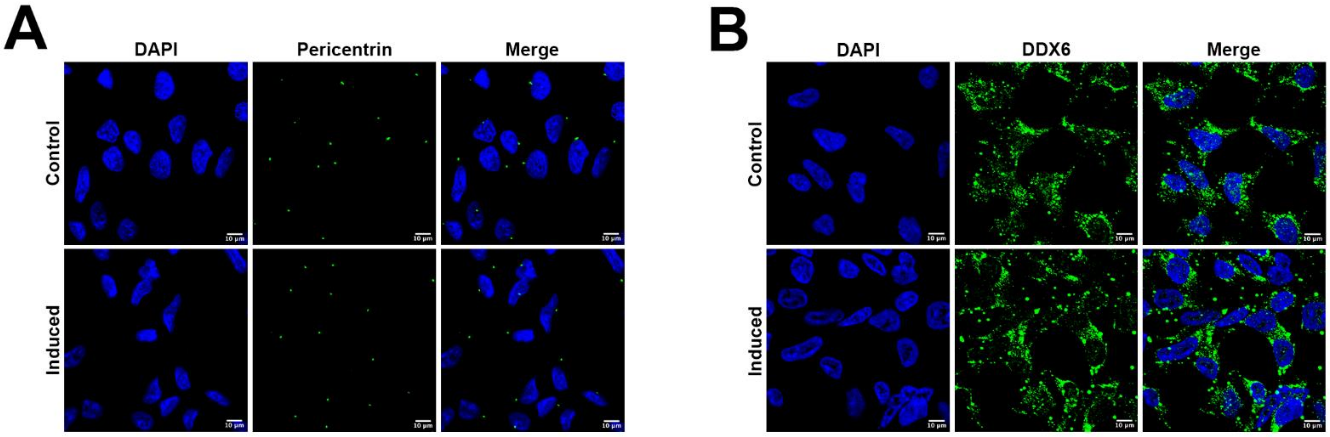

Supplemental Figure 4 – Cytosolic LLPS structures are not affected by polyP. Related to Figure 6. TREx-PPK1 control and cells induced for 48 hours were fixed and stained with specific antibodies for pericentrin (**A**) and P-body helicase DDX6 (**B**). The nucleus was stained with DAPI (blue), and the markers for the centriole and P-bodies were visualised in green.

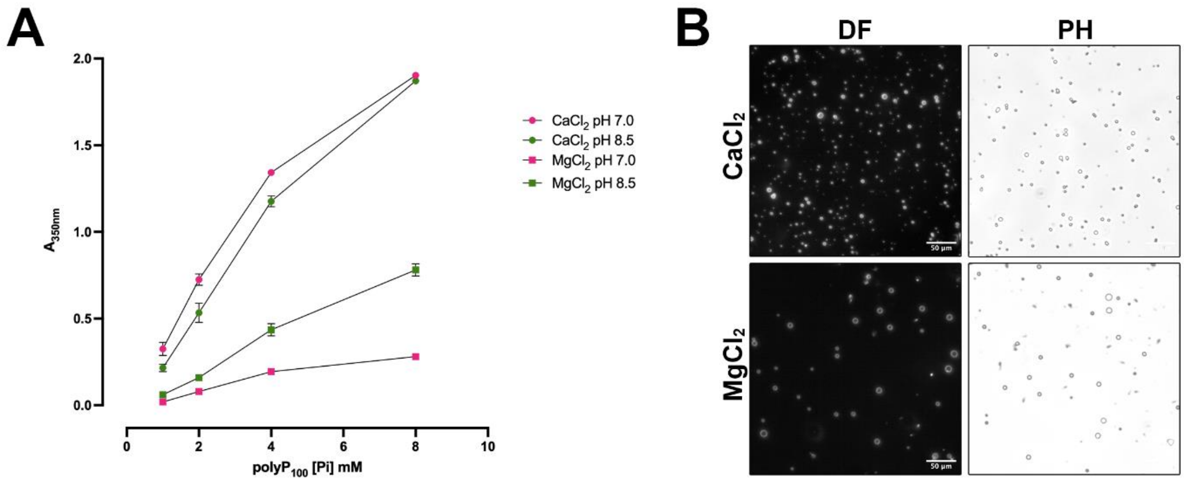

Supplemental Figure 5 – Bivalent cations induce PolyP LLPS phenomena. Related to Figure 7.
Calcium chloride ($CaCl_2$, 10 mM) and magnesium chloride ($MgCl_2$, 10 mM) were incubated in the presence of a 20mM Tris-HCl buffered solution at room temperature with the indicated amount of $polyP_{100}$ for 5 minutes before measuring the turbidity of the solution at 350 nm (**A**). The graph represents the mean ± SEM from three independent experiments. The polyP coacervate (20µl) from the pH 8.5 buffered experiments was spotted on a microscopy slide, and after adding a coverslip, it was observed (**B**) by dark field (DF) and phase contrast (PH) microscopy.
